# α-Xylosidase plays essential roles in xyloglucan remodelling, maintenance of cell wall integrity, and seed germination in *Arabidopsis thaliana*

**DOI:** 10.1093/jxb/erw321

**Published:** 2016-09-07

**Authors:** Takuma Shigeyama, Asuka Watanabe, Konatsu Tokuchi, Shigeo Toh, Naoki Sakurai, Naoto Shibuya, Naoto Kawakami

**Affiliations:** ^1^Department of Life Sciences, School of Agriculture, Meiji University, Higashimita 1-1-1, Tama-ku, Kawasaki 214–8571, Japan; ^2^Graduate School of Biosphere Science, Hiroshima University, Kagamiyama 1-3-2, Higashihiroshima 739–8528, Japan

**Keywords:** Abscisic acid, cell wall, gibberellin, seed dormancy, thermoinhibition, xyloglucan oligosaccharide.

## Abstract

Regulation and maintenance of cell wall physical properties are crucial for plant growth and environmental response. In the germination process, hypocotyl cell expansion and endosperm weakening are prerequisites for dicot seeds to complete germination. We have identified the Arabidopsis mutant *thermoinhibition-resistant germination 1* (*trg1*), which has reduced seed dormancy and insensitivity to unfavourable conditions for germination owing to a loss-of-function mutation of *TRG1/XYL1*, which encodes an α-xylosidase. Compared to those of wild type, the elongating stem of *trg1* showed significantly lower viscoelasticity, and the fruit epidermal cells were longitudinally shorter and horizontally enlarged. Actively growing tissues of *trg1* over-accumulated free xyloglucan oligosaccharides (XGOs), and the seed cell wall had xyloglucan with a greatly reduced molecular weight. These observations suggest that XGOs reduce xyloglucan size by serving as an acceptor in transglycosylation and eventually enhancing cell wall loosening. *TRG1/XYL1* gene expression was abundant in growing wild-type organs and tissues but relatively low in cells at most actively elongating part of the tissues, suggesting that α-xylosidase contributes to maintaining the mechanical integrity of the primary cell wall in the growing and pre-growing tissues. In germinating seeds of *trg1*, expression of genes encoding specific abscisic acid and gibberellin metabolism enzymes was altered in accordance with the aberrant germination phenotype. Thus, cell wall integrity could affect seed germination not only directly through the physical properties of the cell wall but also indirectly through the regulation of hormone gene expression.

## Highlight

Xyloglucan oligosaccharide metabolism by α-xylosidase impacts xyloglucan remodelling, the mechanical integrity of the primary cell wall of growing tissues, cell expansion, and seed germination.

## Introduction

Seed germination is determined by a combination of the growth potential of the embryo and the restrictive potential of the tissues surrounding the embryo (Bewley, 1997). The process of seed germination starts with loss of dormancy and imbibition, and ends with radicle protrusion from the surrounding tissues such as endosperm and testa. Embryo growth for germination is generally brought about by cell expansion without cell division (Bewley, 1997). In *Arabidopsis thaliana* embryos, cell expansion of the lower hypocotyl and the transition zone between the hypocotyl and radicle has been reported to be responsible for embryo growth right through to complete germination (Sliwinska *et al.*, 2009). Extension of the cell is driven by turgor pressure and requires loosening of the cell wall. Hence, the structure and dynamic changes of cell wall components have been considered important in regulating cellular extension. Cellulose microfibrils and hemicellulosic and pectic polysaccharides form a mechanically strong but extensile network in the cell wall (Fry, 1989; Hayashi, 1989; Carpita and Gibeaut, 1993; Cosgrove, 2005). The main hemicellulose in most dicot species is xyloglucan, which cross links cellulose microfibrils in the wall and builds a load-bearing network in the primary cell wall, according to the conventional model. The representative structural unit of xyloglucan is composed of four β-1,4-linked glucosyl residues with three α-1,6-linked xylose side chains (XXXG; according to the nomenclature reported by Fry *et al.*, 1993). Xylosyl residues are often modified by galactose (e.g. XLLG) and further by fucose (e.g. XLFG). Cell wall loosening is thought to be mediated by three proteins: expansin, xyloglucan endotransglycosylase/hydrolase (XTH), and endo-(1,4)-β-glucanase (Cosgrove, 2005). Expansins are activated in acidic pH and induce irreversible wall extension by modifying cross links between cellulose microfibrils and xyloglucan (Cosgrove, 2000). XTH is responsible for the molecular grafting (cleavage and joining) of pre-existing and newly synthesized xyloglucan, and has been postulated to induce both cell wall loosening and strengthening (Fry *et al.*, 1992; Antosiewicz *et al.*, 1997; Nishitani, 1997). Recently, an endoglucanase that hydrolyses both xyloglucan and cellulose, but not xyloglucan- and cellulose-specific endoglucanases, was shown to cause cell wall creep in cucumber (*Cucumis sativus*) hypocotyl (Park and Cosgrove, 2012). This finding and a simulation study of xyloglucan adsorption on cellulose surfaces impacted the conventional model, and resulted in the proposal of a biomechanical hotspot model in which cellulose microfibril contacts are mediated by a subfraction of sandwiched xyloglucans between microfibrils (Zhao *et al.*, 2014; Cosgrove, 2014; Park and Cosgrove, 2015). However, further studies will be required to solve questions and contradictions between the two models.

 The endosperm surrounding an embryo forms a mechanical barrier for germination (Bewley, 1997; Leubner-Metzger, 2003), and endosperm weakening has been proposed as a prerequisite for radicle protrusion in several species (Müller *et al.*, 2006). Cell wall loosening proteins, such as endo-β-mannanase in tomato (*Solanum lycopersicum*) (Nonogaki and Murohashi, 1996), β-1,3-glucanase in tobacco (*Nicotiana tabacum*) (Leubner-Metzger, 2003), and expansins in tomato (Chen and Bradford, 2000), have been reported to be involved in endosperm weakening. In contrast, it has been suggested that *XTH31*, an Arabidopsis XTH gene that is preferentially expressed in the endosperm of germinating seed, is involved in reinforcing the cell wall of the endosperm during seed germination (Endo *et al.*, 2012). Seed germination is thought to be regulated by the balance between abscisic acid (ABA) and gibberellin (GA), and GA stimulates germination by enhancing the growth potential of the embryo and by endosperm weakening. In tomato seeds, GA promotes the expression of cell wall modification enzyme genes that code for endo-β-mannanase, expansin, β-1,3-glucanase, and XTH in the endosperm, and expansin in the embryo; these enzymes could promote germination (Nonogaki *et al.*, 2000; Chen and Bradford, 2000; Chen *et al.*, 2001; Wu *et al.*, 2001; Chen *et al.*, 2002).

 Seed germination is temporally controlled by the combination of an internal factor, dormancy, and environmental factors, such as temperature, light, and oxygen. It has been shown that seed responsiveness to temperature is closely related to the level of dormancy in soil-buried seeds of winter and summer annuals (Baskin and Baskin, 1998). In the case of the winter annual model plant Arabidopsis, the maximum permissive temperature for germination rises gradually during an after-ripening period in summer, but germination is repressed by an environmental temperature higher than the upper limit for germination (thermoinhibition). The seeds germinate in autumn when the temperature falls below the upper limit for germination (Baskin and Baskin, 1998). *De novo* ABA biosynthesis in imbibed seeds was shown to be critical for thermoinhibition of lettuce (*Lactuca sativa*) and winter annual seeds, including Arabidopsis (Yoshioka *et al.*, 1998; Argyris *et al.*, 2008; Toh *et al.*, 2008). In Arabidopsis seeds, high temperature stimulates ABA synthesis by up-regulating expression of the ABA biosynthesis gene *ZEAXANTHIN EPOXYDASE* (*ZEP*) and three of the five *NINE CIS EPOXYCAROTENOID DIOXYGENASE* (*NCED*) genes, *NCED2*, *NCED5*, and *NCED9*; through the action of these ABA genes, high temperatures indirectly suppress GA synthesis. High temperatures do not directly regulate GA biosynthesis and signaling, indicated by the fact that suppression of the GA biosynthesis genes (*GA20ox* and *GA3ox*) and up-regulation of the negative regulator gene of GA signaling (*SPY*) were not observed at high temperatures in ABA-deficient mutant seeds (Toh *et al.*, 2008). 

To study the mechanism of thermoinhibition, we selected five *thermoinhibition-resistant germination* (*trg*) mutants of Arabidopsis (Tamura *et al.*, 2006). One of the three unknown mutants, *trg1*, showed reduced seed dormancy and mild resistance to the GA biosynthesis inhibitor paclobutrazole for germination. *trg1* plants had shorter fruits than the wild type, but plant growth was almost normal.

 In this study, we identified *trg1* as a loss-of-function mutant of the *TRG1/XYL1* gene that has been shown to encode an α-xylosidase (Sampedro *et al.*, 2001; Monroe *et al.*, 2003). This α-xylosidase cleaves xylosyl residue from the non-reducing end of xyloglucan and xyloglucan oligosaccharide (XGO), and has been shown to be a limiting enzyme of XGO degradation (O’Neill *et al.*, 1989; Sampedro *et al.*, 2001). Exogenously applied XGO has been shown to be involved in auxin-induced cell growth (McDougall and Fry, 1990; Takeda *et al.*, 2002; Kaku *et al.*, 2004). *XYL1* loss-of-function mutant alleles were reported to have xyloglucan with reduced fucosylated units, accumulate free XGOs in the growth medium, and show reduced anisotropic growth of fruit and sepal (Sampedro *et al.*, 2010; Günl and Pauly, 2011). However, the impact of XGO accumulation on the physical/mechanical properties of the cell wall, and the connection between the altered XGO metabolism and the growth defects, remain obscure. In this study, we showed over-accumulation of XGO, a size reduction of the xyloglucan chain in growing tissues and germinating seeds, and enhanced cell wall loosening in the elongating flower stem of *trg1*. These mutant properties and tissue-specific expression of *TRG1/XYL1* suggest that α-xylosidase has cell wall and growth modulating functions, and we therefore discuss the function of *TRG1/XYL1* in cell wall loosening and seed germination. We also discuss the possibility of a cell wall integrity signal (Wolf *et al.*, 2012*a*) for the regulation of ABA and GA metabolism gene expression in germinating seeds.

## Materials and methods

### Plant materials and growth conditions

A thermoinhibition-resistant germination mutant of *Arabidopsis thaliana* (L.) Heynh., *trg1-1* (wild type; Wassilewskija, Ws), was screened from the T-DNA insertion library of INRA (Tamura *et al.*, 2006). Col-0 and L*er* accessions were obtained from the Arabidopsis Biological Resource Center (ABRC) and propagated in our laboratory. The seeds of *trg1-2* (transposon inserted gene trap line, GT5839) and *trg1-3* (GABI-Kat T-DNA insertion line, 749G08) were obtained from Cold Spring Harbor Laboratory and the GABI-Kat consortium (Bielefeld University), respectively. *trg1-3* has also been reported as *Atxyl1-2* (Sampedoro *et al.*, 2010) and *axy3.2* (Günl and Pauly, 2011). The seeds were surface sterilized, sown on agar plate, and transferred to a hypotonic culture system as reported previously (Tamura *et al.*, 2006), or they were directly sown and grown on soil (Super Kodoko L, Zenno) in a growth chamber (continuous illumination at 22 °C).

### Germination test

The seeds were harvested at physiological maturity. The seeds were stored in a desiccator for 1.5 months at room temperature for after-ripening. Thirty seeds were imbibed with 300 μl of H_2_O into each well of a 24-well plate at constant temperature in continuous light for 7 days without pre-chilling. Germination in red/far-red-light conditions was tested as follows. The seeds were irradiated with far-red light (740 nm, 1mM/m^2^) or red light (660nm, 6mM/m^2^) from an LED source (Eyela, Tokyo) after pre-imbibition for 1 h at room temperature. The seeds were imbibed at 22 °C for 5 days in complete darkness. Germination was scored as radicle protrusion from both endosperm and testa.

### Molecular mapping

Molecular genetic mapping of *trg1* loci was done as described previously (Tamura *et al.*, 2006). For fine mapping, three molecular markers between 14G4 and KNAT2, TJ-5, FJ-4, and FN-1 were designed in this study (Supplementary Table S1) from sequence polymorphisms between Col-0 and L*er* obtained from the TAIR database (https://www.arabidopsis.org/index.jsp). Recombinants between 14G4 and FN-1 from 1718 F_2_s were selected, and the genotype of *TRG1* loci was determined through the thermoinhibition-resistant phenotype of F_2_ and F_3_.

### Cloning and sequencing

Wild-type *TRG1/XYL1* (At1g68560) and *trg1-1* mutant alleles were amplified and sequenced with primers listed in Supplementary Tables S2 and S3, respectively. The gene sequences with upstream and downstream regions were amplified with PrimeSTAR DNA polymerase (Takara Bio Inc.), and sequenced directly by cycle sequencing with ABI PRISM 3100 Genetic Analyzer (Applied Biosystems). DNA sequences were analysed with GENETYX software (GENETYX Corporation, Tokyo). The sequence data of the *TRG1/XYL1* Ws wild-type allele and *trg1-1* allele were deposited in GenBank (accession numbers LC074691 and LC074692, respectively).

### α-xylosidase activity assay

A preparation of crude extract from seedlings and the α-xylosidase assay were prepared according to Sampedro *et al.* (2010). XXXG (a gift from Dr Kazuhiko Nishitani) was used as a substrate, and released xylose was quantified using the D-Xylose Assay Kit (Megazyme, Ireland).

### Fruit sectioning and microscopy

The developing fruits were harvested at 14 days after flowering from the central part of the flower stem from four independent plants for each genotype. The samples were fixed overnight in 1% formaldehyde, 50 mM phosphate buffer (pH 7.0), and 0.1% Triton X-100. They were then dehydrated through a series of graded ethanol and replaced by resin (Technovit 7100, Kulzer). Cross sections (10 μm) were prepared using a microtome equipped with a disposable knife (SH35W, Feather). The sectioned tissues were stained with 0.5% Toluidine blue and observed with a microscope (Axio Imager A1, Carl Zeiss). The circumference of a carpel (semicircle of a pericarp) was measured from the images using AxioVision software (Carl Zeiss).

### Physical analysis

For the physical analysis, we used ~1-month-old wild-type and *trg1-1* plants, when the second internode reached 3 cm in length. To confirm the elongating part of the stem, the second internodes of five plants were marked every 5 mm, and the intervals between marks were measured after 7 days. The upper- and lower-half of second internode and the base of the flower stem (1.5 cm long each) were cut and boiled in 80% ethanol. Creep-extension analysis was done according to Tanimoto *et al.* (2000). The stem segments were rehydrated with 10mM MES buffer (pH 6.0), and the diameter was measured to obtain the cross-sectional area of the stem. The stem segment was secured between two clamps of a Rheoner creep meter (Yamaden RE-33005, Tokyo). The creep-extension analysis was carried out at room temperature. A constant load of 25 g·mm^−2^ was applied to the stem by driving the lower clamp down at the maximum speed at 0.5 mm·s^−1^. The extension process was recorded by a computer at 0.5 s intervals for 10min. Physical properties were analysed by a computer programme using Burgers’ viscoelastic model to calculate four elastic (E0, E1, E2, E3) and three plastic (η1, η2, η3) parameters involved in the equation below. The curve and the equation are simulated by the Kelvin–Voigt–Burgers’ viscoelastic model:

$$\varepsilon (t)=\frac{{\text{P}}_{0}}{{\text{E}}_{0}}+\frac{{\text{P}}_{0}}{{\text{E}}_{1}}\left(1-{\text{e}}^{-\text{t}/\tau 1}\right)+\frac{{\text{P}}_{0}}{{\text{E}}_{2}}\left(1-{\text{e}}^{-\text{t}/\tau 2}\right)+\frac{{\text{P}}_{0}}{{\text{E}}_{3}}\left(1-{\text{e}}^{-\text{t}/\tau 3}\right)$$

where *ε(t*) is the deformation, P_0_ is the constant load, and τn is the delay time. ηn was calculated by multiplying En by τn.

### Free xyloglucan oligosaccharide extraction and analysis

Ethanol-soluble (75% solution) oligosaccharide was extracted from tissues. The tissues (50 mg) were powdered in liquid N_2_ then homogenized with 3 ml acetone. The homogenate was mixed for 10min and centrifuged, and the pellet was washed three times with 10 ml acetone then washed further with 10 ml acetone overnight with shaking at 4 °C. After centrifugation, the pellet was dried, mixed with 4 ml of 75% ethanol for 10min, and centrifuged, and the supernatant was transferred to micro tubes. Ethanol was evaporated from the extract, and the remaining water-soluble fraction was separated from the pellet after centrifugation. The total sugar content was determined using the phenol-sulfuric acid method. The sample solution (200 μl, containing more than 1 μg of sugar) was mixed with 200 μl of 5% phenol, then mixed with 1 ml of sulfuric acid (96–98%). The absorbance at 490 nm was measured. Glucose was used for a calibration curve.

 Oligosaccharides were separated and identified by matrix-assisted laser desorption/ionization (MALDI) time-of-flight (TOF) MS. The oligosaccharide sample (2 μl) was mixed with 2 μl of the matrix solution (10:1 mixture of 2% 2,5-dihydroxybenzoic and 0.1% NaCl), and 2 μl of the sample mixture was applied to a sample plate and analysed with Voyager DE PRO (PerSeptive Biosystems).

 Quantitative analysis of XXXG was done by high-performance anion-exchange chromatography (HPAEC) with pulsed amperometric detection (PAD). Oligosaccharide samples with 3 μg total sugar (25 μl) were injected into a DX-500 sugar analysis system (DIONEX) equipped with a Carbopac PA-1 anion-exchange analytical column (4×250mm). Oligosaccharides were separated using a linear gradient (B: 0–50%) of solvent A and B (A: 100 mM NaOH; B: 100 mM NaOH/500mM NaOAc) over 30min at a flow rate of 1 ml·min^−1^. The XXXG peak was assigned with standard XXXG (a gift from Dr Kazuhiko Nishitani) and quantified with a calibration curve of standard XXXG using Chromeleon software.

### Extraction of hemicellulose II fraction and gel permeation analysis of xyloglucan

Dry seeds (100 mg) were boiled in 30 ml of methanol, rehydrated, frozen with liquid N_2_ , and powdered in a mortar and pestle. The powdered samples were treated with acetone as described in the section ‘Oligosaccharide extraction’. The washed pellet was dispersed and washed with 10 ml methanol and chloroform (1:1) followed by two washes with 10 ml of ethanol. After washing with de-ionized water, the cell wall material was treated with porcine pancreas α-amylase (2 unit/ml, SIGMA A-6255) in 50 mM NaOAc buffer (pH 6.5) at 37 °C for 3 h to remove starch. The cell wall material was extracted three times with 50 mM EDTA (pH 6.8) at 95 °C for 15min to remove pectins. The material was extracted three times with 3 ml of 4% KOH at 25 °C for 8 h to remove the hemicellulose I fraction. Finally, the residue was extracted three times with 24% KOH and 0.02% sodium borohydride solution at 25 °C for 8 h. The extract (hemicellulose II fraction) was neutralized with acetic acid, dialyzed, and concentrated using a rotary evaporator.

 The xyloglucan content in the hemicellulose II fraction was determined by Kooiman’s iodine staining method (Kooiman, 1960) with a calibration curve of tamarind xyloglucan. The hemicellulose II fraction with 700 μg xyloglucan was dissolved in 50 mM potassium phosphate buffer (pH 7.2) and applied to a gel permeation column (TSKgel G5000PW, 7.5mm × 60cm, TOSOH) equipped in an HPLC system (600E, Waters) with a refractive index detector (830-RI, JASCO). The sample was eluted with 50 mM potassium phosphate buffer (pH 7.2) at a flow rate of 1 ml·min^−1^. Fractions were collected at 30 s intervals. The xyloglucan content in each fraction was determined by Kooiman’s iodine staining method. To determine the molecular mass distribution of xyloglucans, dextrans of 150, 500, and 2500kDa were used as size markers.

### Gene expression analysis by quantitative reverse-transcription PCR

Total RNAs were isolated from tissues, treated with RNase-free DNase, and reverse-transcribed to cDNA for the transcript analysis as described previously (Toh *et al.*, 2008). Quantification of the *TRG1/XYL1* transcript was done by quantitative reverse-transcription (qRT) PCR with fluorescent-labelled nucleotide substrate (Power SYBR PCR mix, ABI). Forward and reverse primer sequences for qRT-PCR are listed in Supplementary Table S4. As reference genes for transcript normalization, we selected At2g20000 (Graeber *et al.*, 2011), At2g28390, At4g34270, and At5g15710 (Czechowski *et al.*, 2005), which showed stable expression in the seeds imbibed at different temperatures in our two-colour microarray analyses (unpublished data with Arabidopsis II 22k array; Agilent). We obtained similar data normalized to the four reference genes. We present the data normalized to the amplification of At4g34270, which showed the most stable expression in our microarray analysis, unless otherwise stated. Reactions were done using the 7500 Fast system (ABI), and the data were analysed using ABI Prism 7700 SDS software (Applied Biosystems). For each sample, the mean value from triplicate qRT-PCRs was adapted to calculate the transcript abundance.

### Transgenic plants

The *TRG1/XYL1* promoter region and the gene containing upstream and downstream regions were amplified from Ws genomic DNA by high-fidelity PCR with specific primers (Supplementary Table S2) and PrimeSTAR DNA polymerase (Takara Bio Inc.). The product was cloned into the Gateway entry vector using the pENTR Directional TOPO Cloning Kit (Invitrogen, K2400-20SP) and transformed One Shot Chemically Competent *Escherichia coli* (Invitrogen, C4040-03). Kanamycin-resistant colonies were selected and plasmid DNA was prepared using the QIAprep Spin Miniprep Kit (Qiagen, 27104). The insertion of the entry plasmids was confirmed by sequencing with primers listed in Supplementary Table S5. The sequence data of Ws *TRG1/XYL1* promoter (*TRG1pro*) and the gene containing upstream and downstream regions (*TRG1pro:TRG1*) used for the transgenic experiments were deposited in GenBank (accession numbers LC074693 and LC074694, respectively). The insertions were transferred to destination vectors with Gateway LR ClonaseII (Invitrogen, 11791-020) and to transformed Competent high *E. coli* DH5α (Toyobo, DNA-903). The *TRG1pro:TRG1* construct was inserted into pGWB1 (Nakagawa *et al.*, 2007) for complementation analysis, and the *TRG1* promoter construct was inserted upstream of the *GUS* gene of pGWB203 (Nakagawa *et al.*, 2007) for expression analysis.

 The *Agrobacterium* C58C1 line was transformed with the destination constructs by electroporation, and the transformants were selected with 50 mg/ml kanamycin and 100 mg/ml rifampicin. *trg1-1* and Ws were transformed using the floral dip method (Clough and Bent, 1998). T_1_ plants were selected with 10 mg/ml Hygromycin B (Roche) and 160 mg/ml Claforan (containing cefotaxime sodium; Sanofi K.K., Tokyo) and the resistant seedlings were transferred to the hypotonic culture system.

### Histochemical β-glucuronidase assay

*TRG1* promoter-driven *GUS* gene expression was observed by whole mount staining of transgenic seeds and seedlings. Tissues were fixed with 90% acetone at −20 °C for 15 min, rehydrated with 100 mM phosphate buffer (pH 7.0), and infiltrated with GUS staining solution [100 mM phosphate buffer (pH 7. 0), 10 mM EDTA, 5 mM potassium ferricyanide, 5 mM potassium ferrocyanide, 0.1% Triton X-100, 0.5 mg/ml X-Gluc (5-bromo-4-chloro-3-indolyl-β-D-glucuronide), GIBCO BRL] under vacuum for 30min. The staining samples were incubated at 27 °C for 1.5 h in darkness, and the reaction was stopped and de-stained with 70% ethanol and a 1:6 mixture of acetic acid and ethanol, before washing with 70% ethanol. The stained tissues were cleared with chloral hydrate solution (trichloroacetaldehyde monohydrate, glycerol, and water in a ratio of 8:1:2) and observed under a differential interference contrast microscope (Axio Imager A1, Carl Zeiss). Images were cropped and the brightness and contrast were adjusted using AxioVision software (Carl Zeiss). We isolated 10 transformants that showed GUS expression, and four independent transformants were used for GUS staining experiments and similar results were obtained.

### Hormone analysis

Acidic hormones were extracted, and ABA and GA_4_ were quantified by liquid chromatography electrospray ionization tandem mass spectrometry (LC-ESI-MS/MS) as described in Preston *et al.* (2009). Hormones were extracted from two independent samples, and similar results were obtained.

## Results

### High temperature-resistant germination mutant *trg1* has a loss-of-function mutation of the α-xylosidase gene

**Fig. 1.**
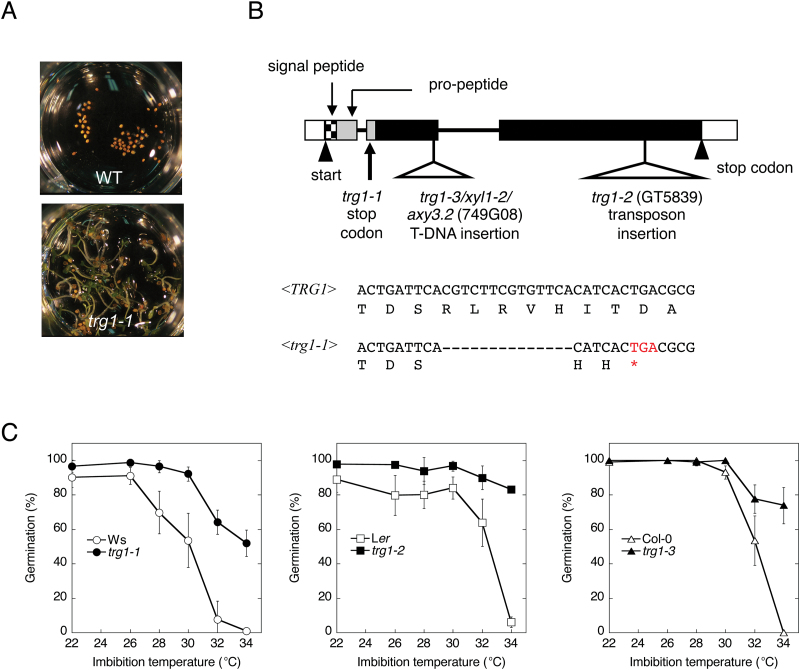
Identification of *trg1* mutation.

We selected five *trg* mutants from T-DNA insertion lines of Arabidopsis (Ws accession) to investigate the mechanism of germination control by high ambient temperature. The seeds of *trg1-1* showed not only thermoinhibition-resistant germination (Fig. 1A) but also reduced dormancy and partial tolerance to the GA biosynthesis inhibitor paclobutrazole (Tamura *et al.*, 2006). The mutation was not tagged with the T-DNA, and had been mapped onto the bottom arm of chromosome 1, between CAPS markers 14G4 and KNAT2 (Tamura *et al.*, 2006; Supplementary Figure S1). We further narrowed down the locus in the 55 kbp region between the two markers (TJ-5 and FJ-4; Supplementary Figure S1; Supplementary Table S1). Seven genes were predicted in this region, but did not include a known germination-related gene. We sequenced the coding region of the six genes except At1g68530, and found a 14bp deletion in the second exon of At1g68560 (Fig. 1B).

(**A**) Thermoinhibition resistance of *trg1-1* seeds. After-ripened Ws (wild type, WT) and *trg1-1* seeds were imbibed at 32 °C for 8 days under continuous light. (**B**) Schematic representation of *TRG1/XYL1* gene and the mutations. Nucleotide and deduced amino acid sequences of *trg1-1* deletion site are shown with corresponding sequences of the wild type (*TRG1*: nucleotides 318–353 from translation start site of the gene sequence). An asterisk shown below the sequence indicates the premature stop codon created by the deletion. (**C**) Effect of imbibition temperature on seed germination of *TRG1/XYL1* alleles under continuous light. The after-ripened seeds were imbibed for 7 days without stratification. Means of three biological replicates with SE are shown for each genotype.

 At1g68560 is known to encode α-xylosidase, and the loss-of-function mutant allele *xyl1-2* almost completely loses α-xylosidase activity against the XGO XXXG (Sampedro *et al.*, 2001; Sampedro *et al.*, 2010). The deletion in the *trg1-1* allele was predicted to create a frame shift and a premature stop codon in the N-terminal pro-peptide region (Fig. 1B). We detected almost no α-xylosidase activity from *trg1-1* seedlings (Supplementary Figure S2), and considered *trg1-1* to be a null allele. To confirm the possibility that the loss of function of *TRG1/XYL1* is responsible for the germination phenotype, we analysed thermoinhibition resistance in two other alleles, *trg1-2* and *trg1-3* (Fig. 1B). *trg1-3* is also named *xyl1-2* (Sampedoro *et al.*, 2010) and *axy3.2* (Günl and Pauly, 2011). Germination of the wild-type seeds was inhibited severely at 34 °C, but the seeds of *trg1-2* and *trg1-3* showed clear thermoinhibition resistance (Fig. 1C). A genetic complementation test and transformation of *trg1-1* with the wild-type gene also support the idea that *TRG1/XYL1* is responsible for the germination phenotype (Supplementary Figure S3).

 The seeds of *trg1* mutants also showed resistance to other unfavourable conditions. There were some variations between alleles, but the seeds showed thermoinhibition tolerance in red-light pulse-induced germination conditions, and the seeds of *trg1-2* showed germination in far-red-light pulse conditions at room temperature (Supplementary Figure S4). These results suggest that *TRG1/XYL1* has a role in germination suppression in response to not only supraoptimal temperature but also other unfavourable conditions for germination.

### The fruit epidermal cells and elongating stem of *trg1* mutant plants have altered texture

**Fig. 2.**
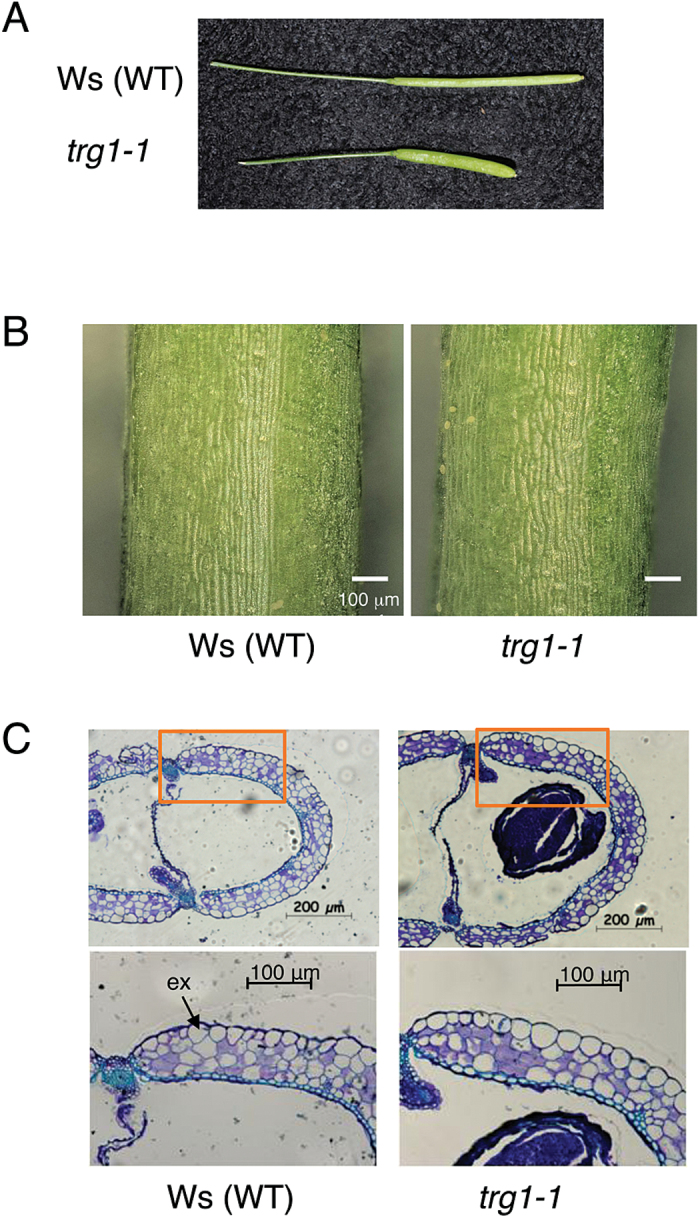
Morphological phenotype of *trg1-1* fruit.

All three alleles of *trg1* produced short and fat fruits (Fig. 2A, Supplementary Figure S3C), as reported by Sampedro *et al.* (2010) and Günl and Pauly (2011). The fruit phenotype was also recovered by transformation with the wild-type *TRG1* gene (Supplementary Figure S3D). Pericarp epidermal (exocarp) cells of *trg1-1* were shorter than those of wild type (Fig. 2B). A cross section of the fruit revealed that *trg1-1* pericarp had horizontally enlarged epidermal cells (Fig. 2C, Table 1). These observations indicate that anisotropic cell expansion was disordered in *trg1-1* pericarp, especially in epidermal cells, and this resulted in shorter fruit length.

(**A**) Fully developed fruit of Ws (wild type, WT) and *trg1-1*. (**B**) Epidermal cell of the central part of the fruit pericarp. Multi-focused images of the fruit epidermis were visualized using a digital microscope (VHX-5000, Keyence). (**C**) Cross sections of the central part of the fruits. The fruit sections (DAF14) were stained with Toluidine blue. ex: exocarp.

**Table 1.** Morphological phenotype of *trg1-1* fruit and exocarp cell

	Wid type (Ws)	*trg1-1*	
Fruit length (mm)	12.2 (0.6)	8.3 (0.5)	* n = 20
Circumference of a carpel (semicircle, μm)	1569 (9)	2163 (118)	* n = 4
Number of exocarp cells in a carpel section	48.5 (2.6)	49.8 (1.7)	n = 4
Average width of a exocarp cell (μm)	32.4 (1.8)	43.5 (2.6)	* n = 4

Number of exocarp cells in a carpel was counted from the stained cross section observed with a microscope, and a typical image is shown in Fig. 2C. The circumference of a carpel (semicircle of a pericarp) was measured from the images using AxioVision software (Carl Zeiss). SD of the biological replicates are shown in parentheses. Asterisks indicate statistical differences between wild-type and mutant values (*P* < 0.05, Student’s *t* test).

 Young *trg1-1* plants frequently displayed a bent flower stem in the growth chamber (Supplementary Figure S5A). The stem showed a gravitropic response, but the movement was delayed by about 1 h (Supplementary Figure S5B). The elongating part of the *trg1-1* stem had a soft texture, which could explain the bending and delayed movement in response to gravity. The pleiotropic effect of *trg1* appeared in seed, fruit, and stem, suggesting that the *trg1* mutation affects the physical properties of the cell wall.

### The elongating part of the *trg1* flower stem has a cell wall with reduced viscoelasticity

To understand the contribution of *TRG1/XYL1* to cell wall physical and mechanical properties, we measured the elasticity and viscosity of the cell wall by creep-extension analysis (Tanimoto *et al.*, 2000). To compare the properties of the cell wall in elongating and non-elongating parts of the stem, we sampled the upper and lower halves of the second internode and the base of the first internode from 1-month-old plants (Supplementary Figure S6A). The length of the second internode had reached 3cm at this stage, and over the next 7 days the upper half elongated further, but the lower half grew very little (Supplementary Figure S6B).

**Fig. 3.**
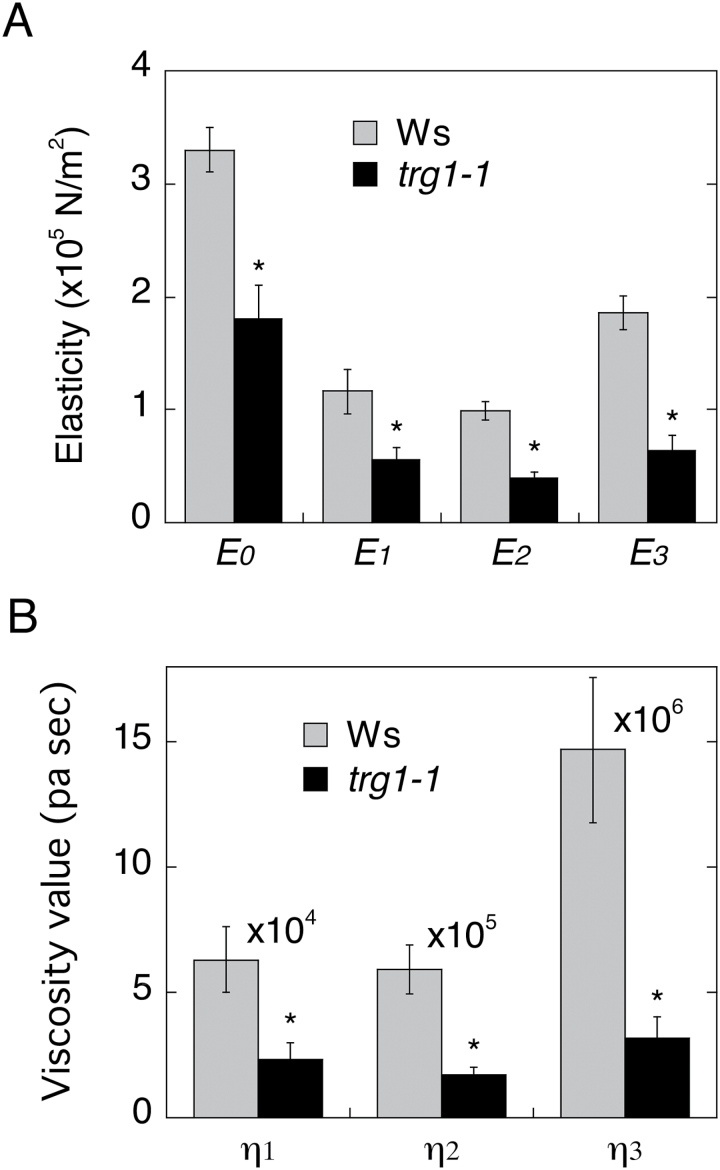
Physical properties of cell wall of elongating stem segments.

 Both the elasticity and viscosity modules of the upper half of the *trg1-1* second internode were lower than those of the wild type (Fig. 3). In contrast, the lower half, which had ceased to elongate, showed similar values between *trg1-1* and wild type, and almost the same values were observed at the bottom part of the inflorescent stem (Supplementary Figure S6C). These results indicate that α-xylosidase activity is required for maintaining the rigidity of the primary cell wall of growing tissues.

The upper-halves (1.5 cm in length) of the elongating second internode (3 cm) were processed for creep-extension analysis. Elasticity (**A**) and viscosity (**B**) values of the wild-type (grey) and *trg1-1* (black) cell wall of the internode are shown. Five stem segments from five plants were used for each genotype, and the SE of the five biological replicates is shown as an error bar. Asterisks indicate statistical differences between wild-type and mutant values (*P* < 0.05, Student’s *t* test).

### Accumulation of xyloglucan oligosaccharides and a reduction of xyloglucan size in *trg1* growing tissues

To understand why the cell wall of the *TRG1* loss-of-function mutant showed reduced viscoelasticity values, we first examined the metabolic changes to xyloglucan in this mutant by analysing free XGOs in *trg1-1* tissues. MALDI-TOF MS analysis of the 75% ethanol-soluble fraction of the cell wall extract identified the potassium adducts of XXXG (m/z 1101) and XXLG/XLXG (m/z 1263) from *trg1-1* mutant fruits (Supplementary Figure S7A). For the quantitative analysis of the free oligosaccharides, the 75% ethanol-soluble fraction was separated by HPAEC with PAD. We found the presence of three peaks specific for *trg1-1* mutant tissue extracts (Supplementary Figure S7B). These peaks were greatly diminished or became undetectable after transformation of *trg1-1* with the wild-type *TRG1/XYL1* gene. The most prominent peak with a retention time of 18.2min was identified as XXXG by co-separation with standard XXXG oligosaccharide.

**Fig. 4.**
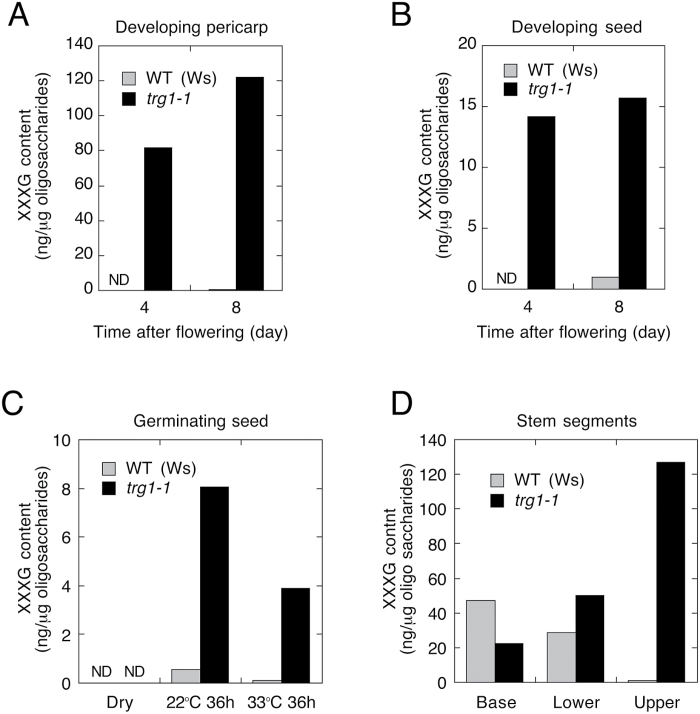
Accumulation of xyloglucan oligosaccharide XXXG in wild-type (WT) and *trg1-1* tissues.

 XXXG was almost below the detection limit in the developing pericarp and seeds of wild-type plants, but was highly concentrated in *trg1-1* tissues (Fig. 4A, B). Only a trace amount of XXXG was detected from germinating wild-type seeds imbibed at 22 °C, whereas a much higher level of XXXG was detected in *trg1-1* mutant seeds (Fig. 4C). XXXG was also detected in germinating *trg1-1* seeds imbibed at 33 °C. The elongating upper half of the internode of *trg1-1* accumulated a high level of XXXG, whereas very little accumulated in wild type (Fig. 4D). The non-elongating lower half of the internode and the base of the wild-type stem accumulated XXXG at the same level as the *trg1-1* mutant.

Free oligosaccharides were extracted from the developing pericarp (**A**), developing seeds (**B**), dry and imbibed seeds (**C**), and stem internode segments (**D**). Oligosaccharides were separated by HPAEC with PAD, and the amount of XXXG was calculated from the peak area. Oligosaccharides were extracted in at least two independent experiments (biological replicates), and the chromatography was repeated twice for each extract. We obtained similar results from the different experiments, and typical data are presented. ND, not detected.

**Fig. 5.**
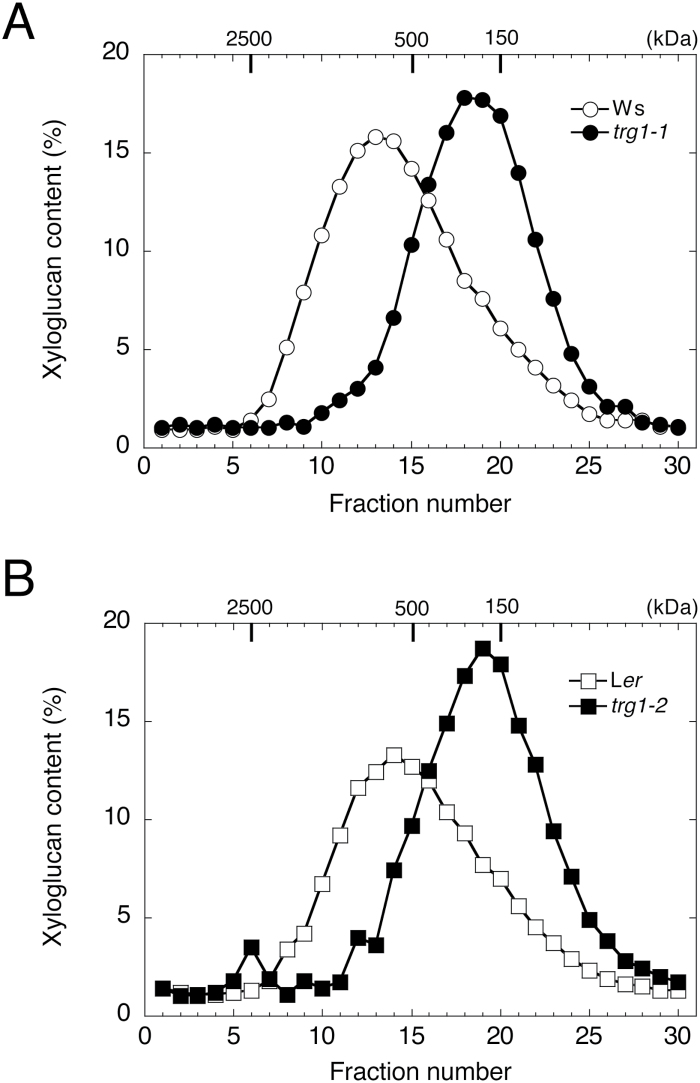
Size distribution of xyloglucan chain in mature dry seeds.

 We also estimated the molecular weight of xyloglucan in the hemicellulose II fraction by HPLC gel permeation analysis. The xyloglucan chains from *trg1-1* and *trg1-2* dry seeds were smaller than those from wild-type seeds (Fig. 5A, B). The estimated molecular mass of xyloglucan in the peak fraction was 660kDa in Ws and 540kDa in L*er*, but was reduced to 240kDa in *trg1-1* and 200kDa in *trg1-2*.

The hemicellulose II fraction (containing 700 μg xyloglucan) was extracted from dry seeds and fractionated by gel permeation chromatography. Xyloglucan chains in each fraction were stained using the Kooiman method, and quantitated by absorbance at 640 nm. The hemicellulose II fraction was prepared in three independent experiments (biological replicates). We obtained similar results from the different experiments, and typical data are presented. (**A**) Xyloglucan extracted from dry seeds of wild type (Ws, open circle) and *trg1-1* (closed circle). (**B**) Xyloglucan extracted from dry seeds of wild type (L*er*, open square) and *trg1-2* (closed square).

### Tissue- and stage-specific expression of *TRG1/XYL1*

**Fig. 6.**
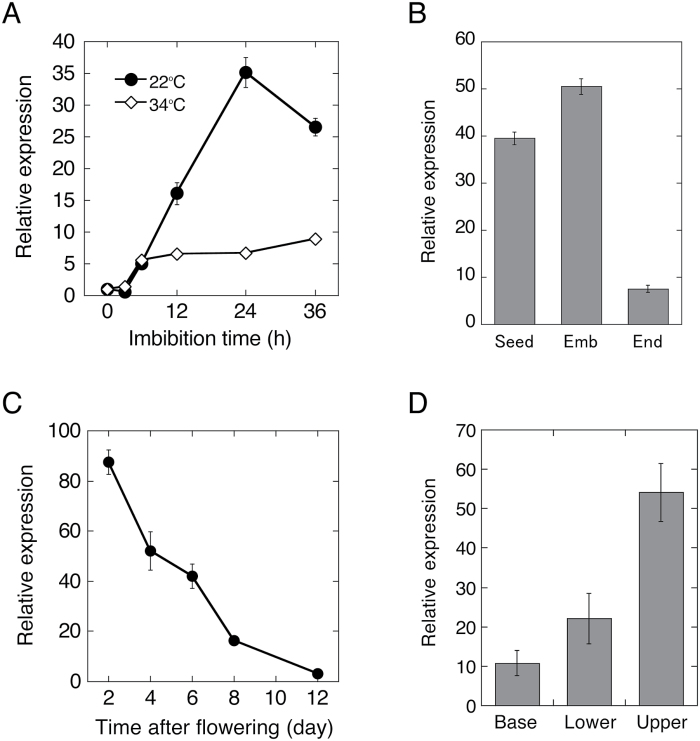
Expression of *TRG1/XYL1* in seeds, fruits, and stem.

*TRG1/XYL1* was reported to be one of the most highly induced genes during seed germination (Cadman *et al.*, 2006). qRT-PCR analysis indicated that the *TRG1/XYL1* transcript level was increased to about 35-fold of dry seed levels during 24 h imbibition at 22 °C (Fig. 6A). *TRG1/XYL1* expression was also induced in thermoinhibited seeds, but the induction was suppressed to about 7-fold of dry seed levels at 24 h after imbibition. The *TRG1/XYL1* transcript was prominent in the germinating embryo, and a relatively low level of expression was detected in the endosperm (Fig. 6B). *TRG1/XYL1* expression was also relatively high in early stages of fruit development (2 days after flowering), and expression was reduced during development (Fig. 6C). In the flower stem, expression was highest in the elongating upper half of the second internode, but relatively low in the lower half and lowest in the base of the stem (Fig. 6D).

Total RNA was prepared from developing fruits, seeds, carpels, and imbibed after-ripened seeds of Ws (wild type). Transcript levels were quantified by qRT-PCR. Values are means of three technical replicates with SDs. RNA extraction and quantification analysis were repeated at least twice with different batches, and we obtained similar results from the different experiments (biological replicates). (**A**) Transcript levels in germinating and thermoinhibited seeds. After-ripened seeds were imbibed at 22 °C (closed circle) or 34 °C (open diamond) under continuous illumination. Relative values to dry seed level are plotted. (**B**) Transcript levels in germinating embryo and endosperm. Imbibed seeds (22 °C for 24 h) were frozen immediately (Seed) or separated to embryo (Emb) and endosperm (End) manually before freezing for RNA extraction. (**C**) Transcript levels in developing fruits. (**D**) Transcript levels in elongating (Upper) and just elongation ceased (Lower) halves of the second internode, and in the non-elongating first internode (Base) of the flower stem. The internode segment positions were the same as those used for the creep-extension analysis and shown in Supplementary Fig. S5A. In B and D, *18S rRNA* was used as reference gene for the transcript normalization.

**Fig. 7.**
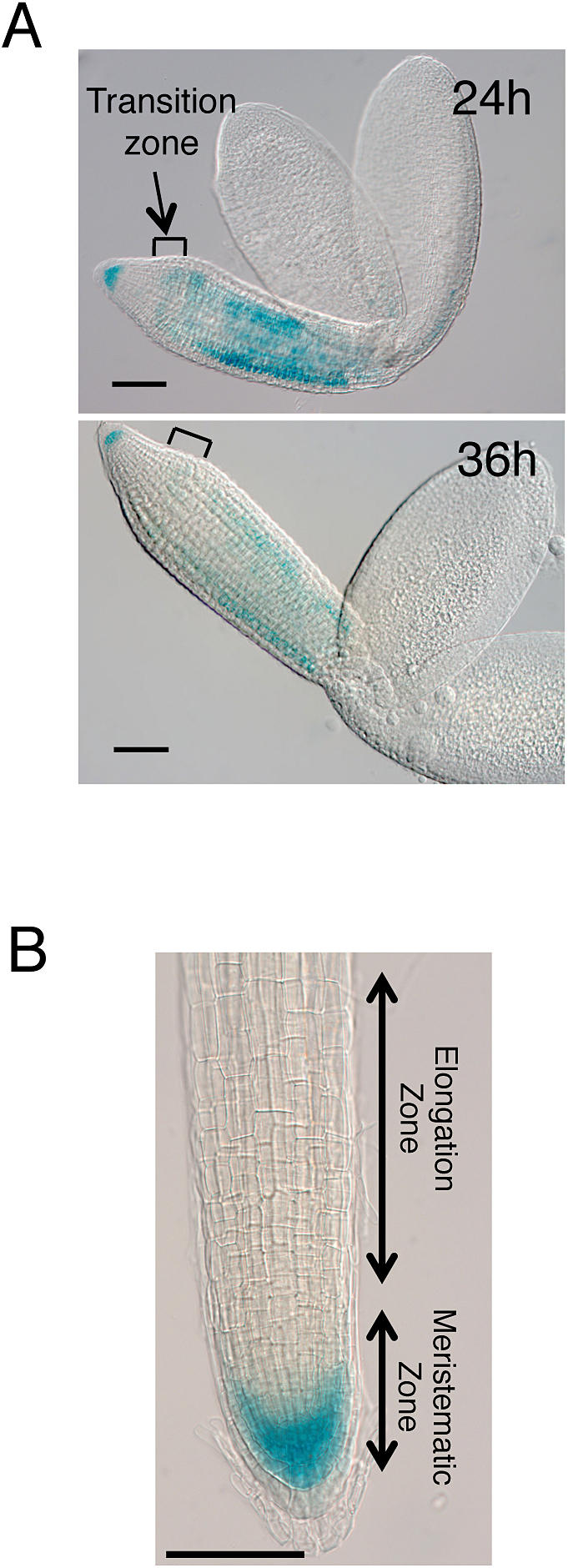
Tissue-specific expression of *TRG1/XYL1* in germinating seeds and roots.

*TRG1pro:GUS* expression analysis indicated tissue-specific expression of *TRG1/XYL1* (Fig. 7). In the 24 h imbibed embryo (before radicle protrusion from endosperm and testa), GUS expression was strongly detected in the radicle tip and upper hypocotyl, but a relatively low level of expression was detected in the lower hypocotyl and the transition zone between the radicle and hypocotyl – which is the region of greatest expansion during germination (Fig. 7A; Sliwinska *et al.*, 2009). After 36 h of imbibition, when the radicle began to appear from the seed coat, GUS staining was reduced, especially in the lower hypocotyl region where cell elongation started. In the root of 7-day-old seedlings, GUS expression was detected in the meristematic zone, but staining was very reduced in the elongation zone (Fig. 7B). These results indicate that *TRG1/XYL1* expression is prominent in growing and pre-growing tissues, but transcript levels are reduced in tissues during growth, especially in the most actively expanding cells.

*TRG1/XYL1* promoter (2060 bp 5′ upstream region containing 5′UTR and start codon)-driven GUS expression was detected after imbibition of the seeds. Imbibed seeds were stained with X-Gluc (5-bromo-4-chloro-3-indolyl-β-D-glucuronide) for 1.5 h, and dissected for microscopy. Four independent transformants were used for GUS staining experiments and similar results were obtained. Bars = 100 μm. (**A**) Germinating seeds imbibed at 22 °C for 24 and 36 h. (**B**) Main root of 7-day-old seedling.

### Abscisic acid and gibberellin metabolism gene expression in *trg1* seeds

**Fig. 8.**
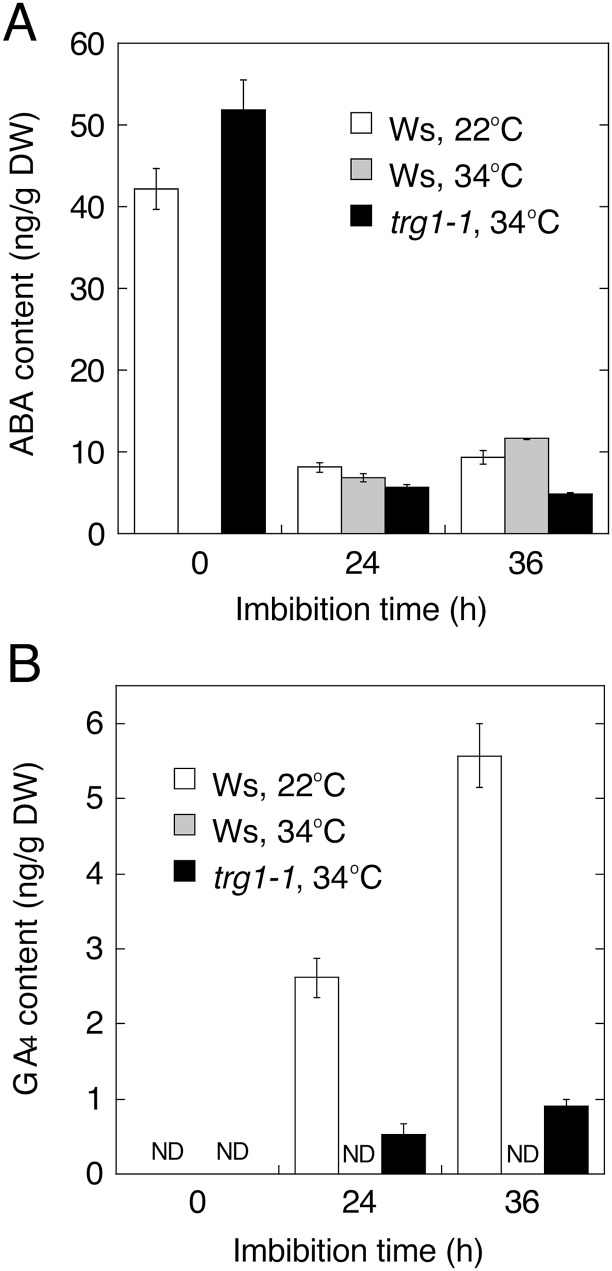
Effect of *trg1* mutation on ABA and GA levels in imbibed seeds.

To see the effect of *trg1* mutation on ABA and GA metabolism, we analysed the hormone levels and expression of ABA biosynthesis and catabolism genes, whose expression is regulated by high temperature in Arabidopsis seeds. Dry mature seeds of *trg1-1* had almost the same level of ABA as wild type (Ws). In Ws seeds, the ABA level continuously decreased during imbibition at 22 °C, but increased from 24 to 36 h of imbibition at 34 °C, as previously observed for Col-0 seeds (Fig. 8A; Toh *et al.* 2008). In *trg1-1* seeds, the ABA level decreased continuously during imbibition at 34 °C, and they had lower levels of ABA than Ws seeds imbibed at 22 °C (Fig. 8A). Ws seeds had increased GA_4_ levels after imbibition at 22 °C, but this increase was completely suppressed at 34 °C (Fig. 8B). In contrast, a relatively low but detectable level of GA_4_ was found in *trg1-1* seeds imbibed at 34 °C (Fig. 8B).

Hormone extraction and quantification analysis were repeated twice with different seed batches, and we obtained similar results from the different experiments. ABA (**A**) and GA_4_ (**B**) levels were quantified by LC-ESI-MS/MS, and typical data from three technical replicates with SE (error bar) are shown. ND, not detected.

**Fig. 9.**
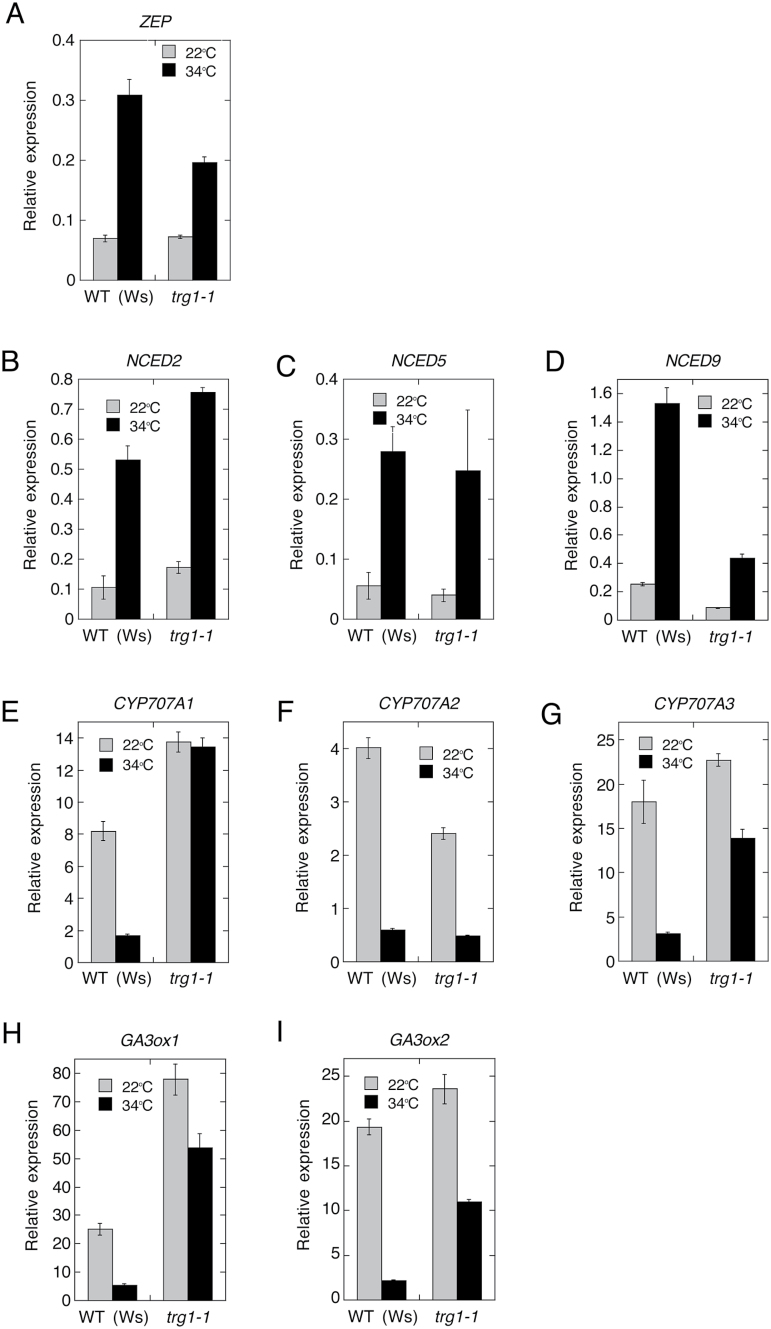
Expression of ABA metabolism and GA biosynthesis genes in imbibed seeds.

 ABA biosynthesis enzyme genes *ZEP*, *NCED2*, *NCED5*, and *NCED9* were up-regulated at supraoptimal high temperatures in wild-type (Ws) seeds, as previously observed in Col seeds (Fig. 9; Supplementary Figure S8; Toh *et al.*, 2008). Expression of *NCED5* was not affected by *trg1-1* mutation, but *ZEP* and *NCED9* expression was suppressed in *trg1-1* seeds, especially at high temperatures (Fig. 9; Supplementary Figure S8). *ZEP* is a single gene that encodes zeaxanthin epoxidase (Audran *et al.*, 2001), and *NCED9* plays a major role in high temperature-induced ABA synthesis and thermoinhibition among the five *NCED*s (Toh *et al.*, 2008); thus, the suppression of these two genes could be responsible for the reduced ABA level in *trg1-1* seeds imbibed at 34 °C. In contrast to ABA biosynthesis genes, expression of the ABA catabolism genes *CYP707A1*, *CYP707A2*, and *CYP707A3* was suppressed at high temperatures in Ws seeds (Fig. 9; Supplementary Figure S8). Expression of *CYP707A2*, which plays a major role in ABA catabolism in germinating seeds (Kushiro *et al.*, 2004), was almost normal in *trg1-1* seeds (Fig. 9; Supplementary Figure S8). However, expression of *CYP707A1* and *CYP707A3* was higher in *trg1-1* seeds imbibed at both room temperature and high temperatures than in Ws seeds imbibed at room temperature (Fig. 9; Supplementary Figure S8). The enhanced expression of *CYP707A3* was also observed shortly after the start of imbibition. These results indicate that both the biosynthesis and catabolism of ABA were influenced by the *TRG1/XYL1* loss-of-function mutation irrespective of the imbibition temperature, and that reduced biosynthesis and enhanced catabolism of ABA could have some impact on the thermoinhibition-resistant and far-red-light-resistant germination and reduced dormancy phenotypes of *trg1*.

Transcript levels were quantified by qRT-PCR. Relative values to dry seed level are plotted. RNA was extracted from seeds imbibed for designated time to quantify each gene expression; *ZEP*, 36 h (**A**); *NCED2*, 24 h (**B**); *NCED5*, 36 h (**C**); *NCED9*, 36 h (**D**); *CYP707A1*, 36 h (**E**); *CYP707A2*, 6 h (**F**); *CYP707A3,* 36 h (**G**); *GA3ox1*, 6 h (**H**), *GA3ox2*, 24 h (**I**). Values are means of three technical replicates with SDs. RNA extraction and quantification analysis were repeated four times with different seed batches, and we obtained similar results from the different experiments.

 Expression of *GA3ox1* and *GA3ox2*, which encode key enzymes of active GA biosynthesis, was also affected by *trg1* mutation (Fig. 9; Supplementary Figure S8). Both genes were down-regulated at supraoptimal high temperatures in the wild-type seeds, but the suppression was greatly relieved in *trg1-1* seeds. Expression of *GA3ox1* was also enhanced in *trg1-1* seeds imbibed at 22 °C (Fig. 9; Supplementary Figure S8). These results suggest that the *trg1* mutation affects specific genes of ABA and GA metabolism, and that altered metabolism of the two main germination-related hormones is also responsible for the germination phenotype of *trg1* seeds.

## Discussion

### *TRG1/XYL1* is a germination suppressor

We identified a high temperature-resistant germination mutant, *trg1-1*, as having a loss-of-function mutation of *TRG1/XYL1* (At1g68560), which encodes an α-xylosidase. Sampedro *et al.* (2010) reported that T-DNA insertion alleles of *XYL1*, *xyl1-1* in the Ws background, and *xyl1-2* in the Col background almost completely lack α-xylosidase activity. We used the *xyl1-2* allele in our study (designated as *trg1-3*) and it showed a similar germination response and fruit phenotype to *trg1-1* and *trg1-2* (Fig. 1 and Supplementary Figure S3). The Col and Ws genomes have close paralogues of *XYL1*, but it is thought to be a pseudogene in both genomes (Sampedro *et al.*, 2010). Our sequence analysis showed that the *trg1-1* mutation created a premature stop codon in the N-terminal pro-peptide region (Fig. 1B), and α-xylosidase activity was not detected in *trg1-1* (Supplementary Figure S2). We confirmed that α-xylosidase has a role in germination by analysing the high temperature-resistant germination phenotype of *trg1* alleles of different genetic backgrounds, *trg1-2* and *trg1-3/xyl1-2* (Fig. 1C; Supplementary Figure S4A), and by complementation analyses (Supplementary Figure S3). Seed germination and the dormancy phenotypes of *trg1* mutant alleles indicate that *TRG1/XYL1* is not specific for germination suppression in response to high temperature, but it works as a general suppressor of seed germination in Arabidopsis (Supplementary Figure S4).

### Contribution of α-xylosidase to physical properties of the cell wall and to growth

The *trg1* mutation was pleiotropic; in addition to the germination phenotypes, *trg1* alleles had short fruit as reported by Sampedro *et al.* (2010) and Günl and Pauly (2011), and also showed bending and delayed gravitropic responses in the flower stem (Supplementary Figure S5). The reduced viscoelasticity of the elongating part of the internode and the almost normal viscoelastic value of the non-elongating part of the internode of *trg1-1* indicate that α-xylosidase is required to maintain the physical strength of the primary cell wall in growing tissues (Fig. 3, Supplementary Figure S6). We could not measure the physical properties of the seed cell wall because of its small size, but cell wall over-loosening could also take place in *trg1-1* seed tissues, which causes seeds to germinate under unfavourable light and temperature conditions and to be less dormant (Fig. 1, Supplementary Figure S4).

 Seed germination is determined by the balance between the growth potential of the embryo and the barrier potential of the embryo surrounding tissues such as the endosperm and testa. The majority of *TRG1/XYL1* transcripts in seeds were detected in the embryo (Fig. 6B). In the imbibed seed embryo, tissue- and cell type-specific expression of *TRG1/XYL1* could contribute to the control of embryo growth potential for germination. The expansion of cells in the lower hypocotyl and the transition zone between the hypocotyl and radicle was reported to be responsible for embryo growth through to complete germination (Sliwinska *et al.*, 2009). *TRG1/XYL1* promoter-driven GUS expression analysis indicated that *TRG1/XYL1* expression is abundant in the root tip and upper hypocotyl, but relatively low in the most actively elongating cells in the embryo of the germinating seed (Fig. 7A). In the root of the seedling, *TRG1/XYL1* expression was high in the meristematic zone, but very low in the elongation zone (Fig. 7D). The suppression of *TRG1/XYL1* expression could allow cell wall loosening in the most actively elongating cells, and high levels of *TRG1/XYL1* expression gives appropriate physical/mechanical strength to the cells in the pre-growing part of the growing tissues.

 Relatively low but detectable expression of *TRG1/XYL1* in the endosperm (Fig. 6B) suggests that α-xylosidase also works in the endosperm and modulates seed germination. Suppression of *TRG1/XYL1* expression in the germinating endosperm could allow cell wall loosening in combination with other endosperm-weakening proteins, such as endo-β-mannanase, β-1,3-glucanase, and expansins (Nonogaki and Murohashi, 1996; Chen and Bradford, 2000; Leubner-Metzger, 2003). Testa is a maternal tissue, and F_1_ seeds from the reciprocal cross between *trg1-1* and Ws showed no thermoinhibition tolerance, similar to the wild-type seeds (Tamura *et al.*, 2006). This suggests that the activity of *TRG1/XYL1* in the testa could have little effect on seed germination.

**Fig. 10.**
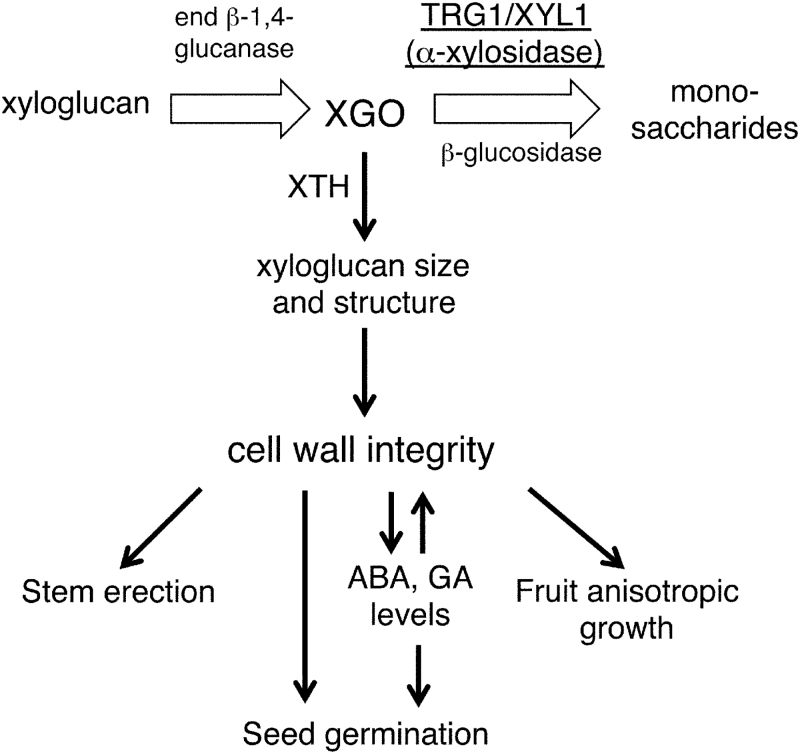
A model of the function of α-xylosidase and its substrate XGO in primary cell wall loosening and growth of the tissues.

 Our study indicates that α-xylosidase could be involved not only in the turnover and recycling of sugars from xyloglucan in the growing tissues, but also in modulating the mechanical properties of the primary cell wall and tissue growth by tuning the XGO levels (Fig. 10). Our gene expression analyses suggest that the major contribution of *TGR1/XYL1* to seed germination is to modulate embryo growth potential by affecting the physical properties of the cell wall. The subcellular localization and enzyme activity of TRG1/XYL1 protein, however, are also important to understand the function and contribution of α-xylosidase to seed germination. α-xylosidase is an apoplastic protein secreted from the cells (Sampedro *et al.*, 2001, Casasoli *et al.*, 2008). It has been suggested that α-xylosidase removes xylosyl residues from the non-reducing end of XGOs produced by endoglucanases during cell growth, thus allowing further degradation of XGO (O’Neill *et al.*, 1989; Fanutti *et al.*, 1991). The aberrant accumulation of XGO in the *TRG1/XYL1* loss-of-function mutant tissues supports this function (Fig. 9, Sampedro *et al.*, 2010). We detected abundant *TRG1/XYL1* expression in the germinating radicle tip and upper hypocotyl (Fig. 7A), and the produced α-xylosidase could attack XGO produced not only in the embryo but also in the surrounding endosperm. XGO could diffuse easily from the endosperm to embryo and *vice versa*, and it is also possible that α-xylosidase itself moves from the secreted site to neighbouring tissues. An evaluation of embryo growth potential by image-based analysis has been reported, which measured embryo size increases in different osmotic media (Voegele *et al.*, 2012).

Kaida *et al.* (2010*a*) reported the possibility of α-xylosidase enzyme activity control by purple acid phosphatase (PAP). They found that, in tobacco, α-xylosidase in the cell wall and culture medium was phosphorylated. They showed that Arabidopsis α-xylosidase produced in yeast cells could be dephosphorylated and deactivated by PAP. In addition, over-expression of *PAP* in tobacco cells decreased α-xylosidase activity in the cell wall and in the culture medium, resulting in the accumulation of XGOs. In Arabidopsis, expression of *PAP* genes such as *AtPAP10* and *AtPAP12* has been reported to be up-regulated during seed germination (Suzuki *et al.*, 2003; Bassel *et al.*, 2008). Wang *et al.* (2014) reported that AtPAP12 and AtPAP26 are major intracellular and secreted acid phosphatases in Arabidopsis whereas AtPAP10 is mainly a secreted acid phosphatase. These apoplastic phosphatases could have some role in modulating α-xylosidase activity during seed germination. Further studies on protein localization and enzyme activity in combination with an embryo growth potential analysis could provide a more clear picture of α-xylosidase function in seed germination.

### Molecular function of α-xylosidase on cell wall loosening, growth, and development

In this study, we found that xyloglucan in the hemicellulose II fraction extracted from *trg1-1* and *trg1-2* seeds had a greatly reduced molecular weight (Fig. 5). XGOs can reduce xyloglucan size by acting as an acceptor of the cleaved xyloglucan chain catalysed by XTH, as suggested by Fry *et al.* (1992) and Monroe *et al.* (2003), and discussed below. Takeda *et al.* (2002) reported that exogenously applied XXXG was integrated into pea (*Pisum sativum*) stem segments, and this incorporation was inhibited by anti-XTH antibody. In the pea stem segments, XXXG solubilized xyloglucan from the cell wall, and enhanced the extensibility of the cell wall. Kaku *et al.* (2004) reported that exogenously applied XGOs enhanced the extensibility of epidermal tissue strips peeled from adzuki bean (*Vigna angularis*) epicotyl in a dose-dependent manner, and the enhancement was XTH dependent. They also reported a reduction in the size of wall-bound xyloglucan after the addition of XGOs. This size reduction was XGO and XTH dependent. In tobacco suspension culture cells, exogenously applied XXXG reduced the size of the xyloglucan, enhanced cell expansion and cell division, and changed the cell shape from cylindrical to spherical (Kaida *et al.*, 2010*b*).

 These reports strongly support our idea on the function of endogenous XGO and α-xylosidase in growth and development (Fig. 10). In actively growing tissues, XGOs produced by endoglucanases are degraded by glycosidases including α-xylosidase. An excess amount of XGO inhibits grafting of the xyloglucan chain and reduces its size, because XGOs can be incorporated into newly digested xyloglucan instead of the xyloglucan chain by the endotransglycosylase reaction of XTH. Following the conventional model (Fry, 1989; Hayashi, 1989; Carpita and Gibeaut, 1993; Cosgrove, 2005), xyloglucan is predicted to cross link cellulose microfibrils and build a load-bearing network in the primary cell wall. The inhibition of xyloglucan grafting could reduce the viscoelasticity of the cell wall, and enhance the extensibility of the cell. We have no direct evidence, however, on the causal relationship between xyloglucan length and cell wall loosening. If xyloglucan does not work as a tether between the separate cellulose microfibrils as proposed in the biomechanical hotspot model (Park and Cosgrove, 2015), only the size reduction of xyloglucan can not explain the reduction in the viscoelasticity of the cell wall. It could also be possible that a combination of the level of XGOs, the xyloglucan size, and/or structure of xyloglucan affect the mechanical properties of the primary cell wall by unknown mechanisms. The *trg1* mutant allele, *xyl1-2*/*axy3.1* (*trg1-3* in this study) has been shown to have xyloglucan with an increased proportion of non-fucosylated xyloglucan structural units (Sampedro *et al.*, 2010; Günl and Pauly, 2011). Günl and Pauly (2011) also showed that less-fucosylated xyloglucans were less tightly associated with other cell wall components. Xyloglucan with a reduced fucosylation ratio has also been shown to have a reduced ability to bind to cellulose microfibril (Hayashi *et al.*, 1994). Thus, α-xylosidase could also contribute to cell wall extensibility by modulating the structure of xyloglucan.

 A reduction in the viscoelasticity of the cell wall makes the elongating stem soft, but the degradation of XGO by α-xylosidase maintains cell wall strength and keeps the stem erect. In the epidermis of fruit pericarp, XGO accumulation makes the cells spherical rather than cylindrical, as observed in tobacco suspension culture cells (Kaida *et al.*, 2010*b*), but α-xylosidase guarantees cylindrical elongation of the epidermal cells by XGO catabolism. During seed germination, XGO could be digested most actively in the radicle and upper hypocotyl, and the suppression of α-xylosidase activity could enhance the extensibility of the lower hypocotyl and transition zone cells for germination. *TRG1/XYL1* could also have a suppressive role in endosperm weakening by preventing over-loosening of the endosperm cells. Recently, Arabidopsis *XTH31,* which is expressed in the endosperm of germinating seed, was shown to work as a germination suppressor (Endo *et al.*, 2012). α-xylosidase could modulate endosperm cell wall properties by enhancing XTH31 molecular grafting activity during seed germination (Fig. 10).

### Do the mechanical properties of the primary cell wall affect seed germination directly and/or indirectly through the regulation of abscisic acid and gibberellin levels?

The metabolism of the two major phytohormones involved in seed germination was also affected by *trg1* mutation in the seeds. Reduced up-regulation of ABA biosynthesis genes, and de-suppression of ABA catabolism and GA biosynthesis genes could suppress an increase in ABA levels and induce an increase in GA levels in *trg1-1* seeds at high temperature (Figs 8 and 9). This disordered expression of hormone genes may not be the result of germination because the differential expression between *trg1-1* and wild type was observed at early times of seed imbibition; at 3 h of imbibition for *CYP707A3* and at 6 h of imbibition for *GA3ox1* (Fig. 9D, E). These observations suggest that the altered metabolism of the two main germination-related hormones, ABA and GA, is also responsible for the germination phenotype of *trg1* seeds.

 The mechanical properties of the cell wall itself could directly affect cell expansion and tissue growth as discussed, but it is also possible that the cell wall properties and/or compositions act as a signal for the modulation of ABA and GA metabolism in the seeds. The aberrant suppression of *NCED9* and the up-regulation of *CYP707A1*, *CYP707A3*, and *GA3ox1* were also observed in *trg1-1* seeds imbibed at room temperature (Fig. 9). This disordered expression was not observed for all the thermoinhibition-related hormone metabolism genes, and expression of *NCED2*, *NCED5*, and *CYP707A2* genes was almost normal in *trg1-1* seeds (Supplementary Figure S8). The gene-specific effect of the *trg1* mutation suggests the existence of specific signalling or of a specific regulatory pathway in response to the changes in the seed cell wall. The regulation and maintenance of the cell wall physical properties are crucial for growth and for environmental responses, and the presence of a system to monitor cell wall integrity has long been inferred (Wolf *et al.*, 2012*a*). In support of this cell wall signal hypothesis, perturbation of cellulose synthesis by genetic mutation or pharmacological interference brings about a variety of secondary effects, including a compensatory response (Seifert and Blaukopf, 2010). Suppressor mutant screening for cellulose-deficiency (*procuste1-1*) and for over-expression of pectin methylesterase inhibitor protein identified a receptor-like kinase (THESEUS1) and a receptor-like protein (RLP44), respectively, as strong candidates for sensors of cell wall integrity (Wolf *et al.*, 2012*b*; Wolf *et al.* 2014).

 ABA and GA are well known to have critical roles in cell wall remodelling for germination by regulating the expression of cell wall modification genes (Nonogaki *et al.*, 2000; Chen and Bradford, 2000; Chen *et al.*, 2001; Wu *et al.*, 2001; Chen *et al.*, 2002). Our present study suggests that an inverse regulation process is also working in seed germination, in which cell wall properties modulate ABA and GA metabolism by regulating the expression of biosynthesis and catabolism genes. This could form a positive feedback system to provide growth potential for the embryo and to weaken the endosperm, both of which are essential for successful germination. This feedback system could also work negatively on the germination of seeds in unfavourable conditions, and could constitute a part of the regulation system of germination in response to environmental signals.

## Supplementary data

Supplementary data are available at *JXB* online.

Figure S1. Molecular mapping of *trg1* locus.

Figure S2. α-xylosidase activity in *trg1* alleles.

Figure S3. Complementation analyses of *trg1* alleles.

Figure S4. Seed germination of *TRG1* alleles in red-light and far-red-light conditions.

Figure S5. Bending and gravitropic movement of *trg1-1* flower stem.

Figure S6. Elongation of second internode halves and physical properties of non-elongating part of the stem.

Figure S7. HPLC and MALDI/TOF MS analyses of free oligosaccharides.

Figure S8. Expression time course of ABA metabolism and GA biosynthesis genes in imbibed seeds.

Table S1. Molecular markers used for *trg1* mapping.

Table S2. Primers for *TRG1/XYL1* (At1g68560) and *trg1-1* mutant allele cloning by PCR.

Table S3. Primers for *TRG1/ XYL1* and *trg1-1* sequencing.

Table S4. Primers for gene expression analyses with qRT-PCR.

Table S5. Primers for the confirmation of clone sequence for complementation and tissue-specific gene expression analyses.

Supplementary Data
