# Supplementary material for: α-Xylosidase plays essential roles in xyloglucan remodelling, maintenance of cell wall integrity, and seed germination in Arabidopsis thaliana
Source: J Exp Bot. 2016 Sep 7;67(19):5615–29. doi: 10.1093/jxb/erw321 (PMC5066485; doi:10.1093/jxb/erw321)
Supplement: Supplementary Data [file supp_67_19_5615__index.html]

α-Xylosidase plays essential roles in xyloglucan remodelling, maintenance of cell wall integrity, and seed germination in Arabidopsis thaliana — α-Xylosidase plays essential roles in xyloglucan remodelling, maintenance of cell wall integrity, and seed germination in Arabidopsis thaliana — Supplementary Data 

# α-Xylosidase plays essential roles in xyloglucan remodelling, maintenance of cell wall integrity, and seed germination in *Arabidopsis thaliana*

## Supplementary Data

Data files

- Supplementary\_Figures\_S1\_S8.pdf - Supplementary Data
- Supplementary\_Tables\_S1\_S5.pdf - Supplementary Data
