# Supplementary material for: α-Xylosidase plays essential roles in xyloglucan remodelling, maintenance of cell wall integrity, and seed germination in Arabidopsis thaliana
Source: J Exp Bot. 2016 Sep 7;67(19):5615–29. doi: 10.1093/jxb/erw321 (PMC5066485; doi:10.1093/jxb/erw321)
Supplement: Supplementary Data [file supp_erw321_Supplementary_Figures_S1_S8.pdf]

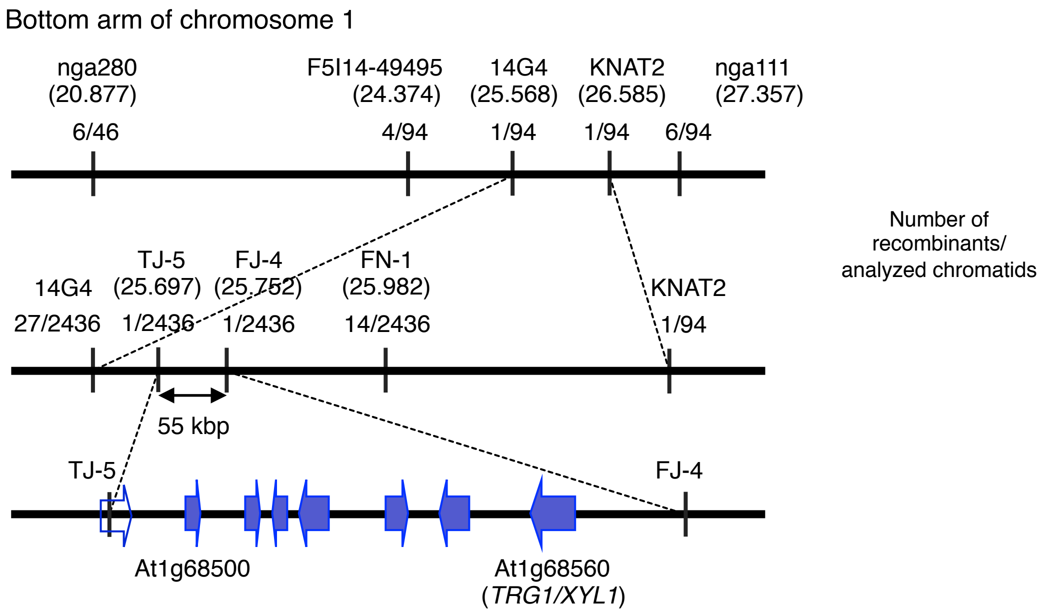

Supplementary Figure S1. Molecular mapping of *trg1* locus. Molecular markers used in this study is listed in Supplementary Table 1. Positions (Mbp) of the markers are indicated in parenthesis. Number of recombinant chromatids found in total number of chromatids tested is shown for each marker.

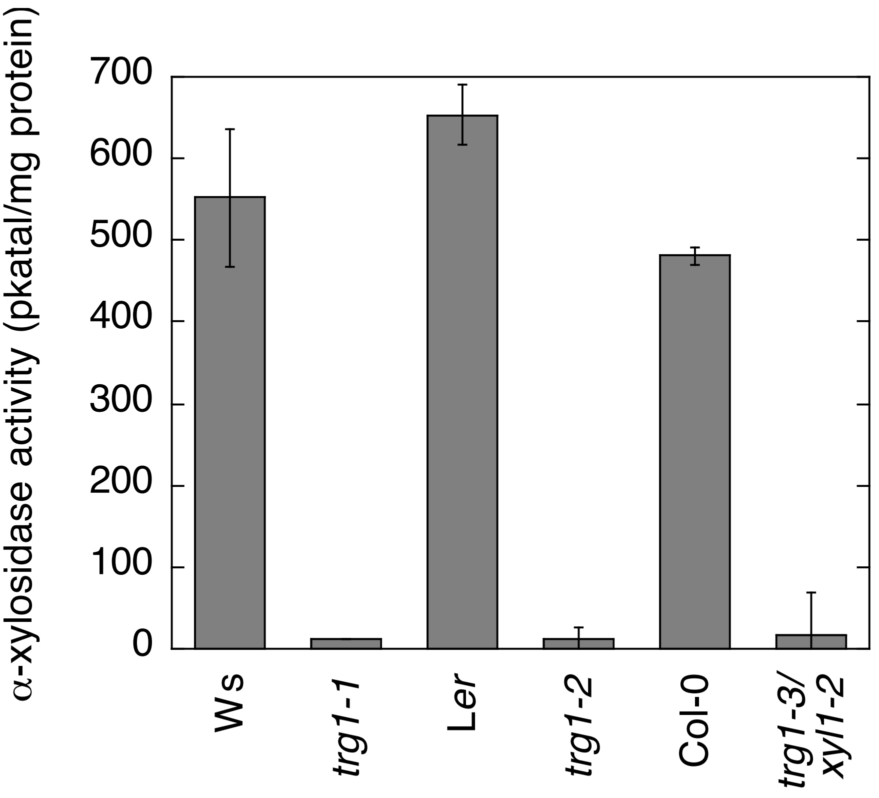

Supplementary Figure S2. α-xylosidase activity in *trg1* alleles.

Crude protein samples were extracted from shoots of 10 day old seedlings. Structure of the mutant alleles are shown in Figure 1B. *trg1-3* is also named *as xyl1-2/axy3.2*.

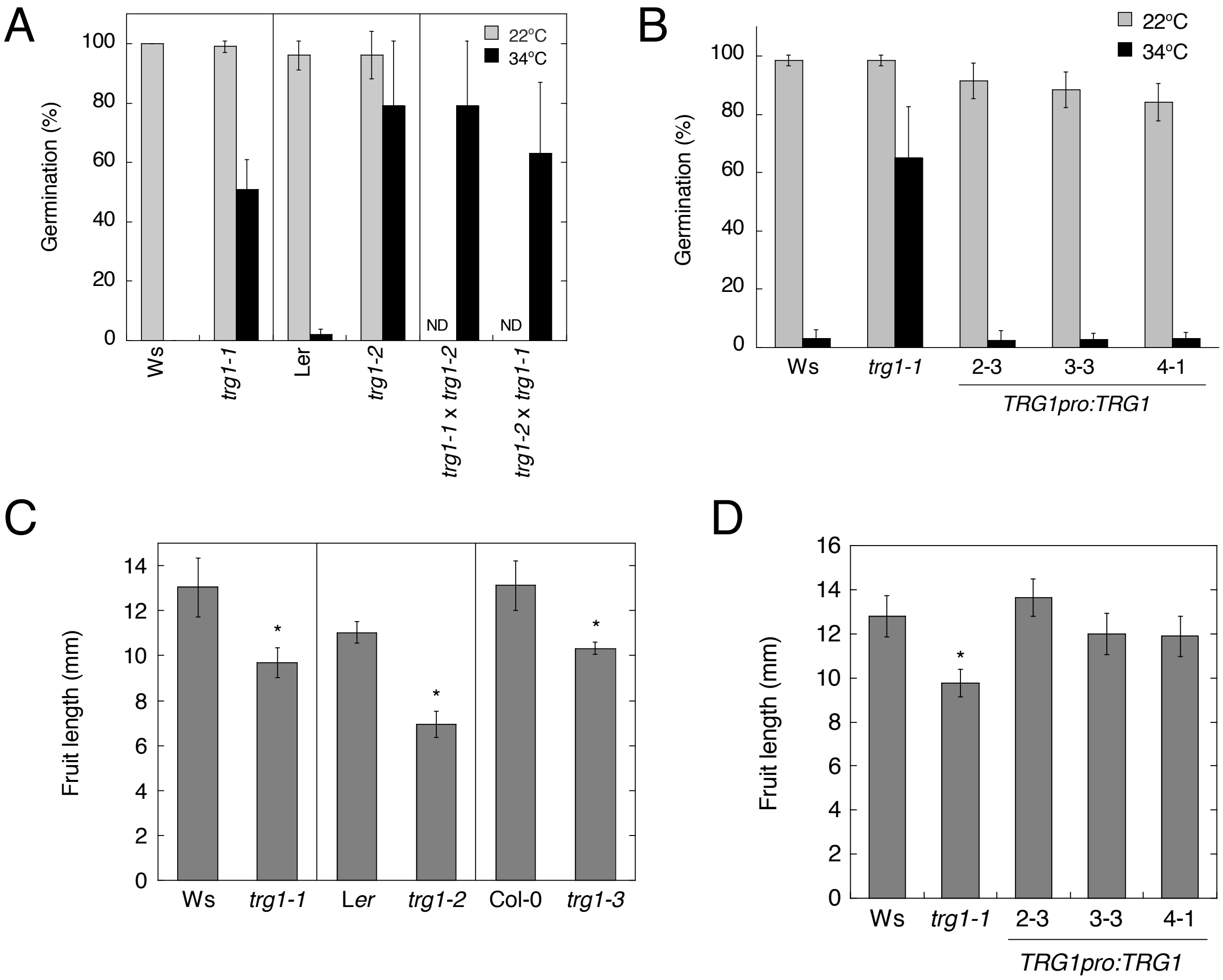

Supplementary Figure S3. Complementation analyses of *trg1* alleles.

(A) Genetic complementation of thermoinhibition resistant germination phenotype. F_1 seeds from reciprocal cross between *trg1-1* and *trg1-2* were used for thermoinhibition assay with the parents and their wild type seeds. After-ripened seeds were imbibed at 22°C (gray) or at 34°C (black) for 7 days under continuous light.

(B) Complementation of thermoinhibition resistant germination phenotype by transformation of *trg1-1* with wild type *TRG1* gene construct. *trg1-1* was transformed with wild type *TRG1* gene construct containing 2kbp upstream and 1kbp downstream regions of the gene. After-ripened seeds were imbibed at 22°C (gray) or at 34°C (black) for 7 days under continuous light.

In (A) and (B), means of three biological replicates with SE are shown for each genotype.

(C) Short fruit length phenotype of *trg1* mutant alleles.

(D) Complementation of short fruit phenotype by transformation of *trg1-1* with wild type *TRG1* gene construct.

In (C) and (D), five fruits from a central part of the flower stem of ten plants were used for the measurement with a micrometer caliper. Means of ten biological replicates with SE are shown for each genotype. Asterisks indicate statistical differences from wild type value ($P<0.05$, Student's *t* test).

A

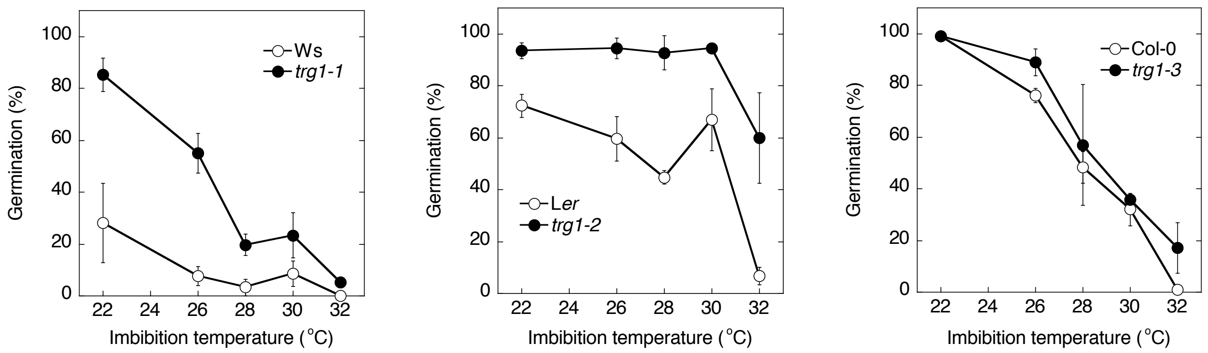

B

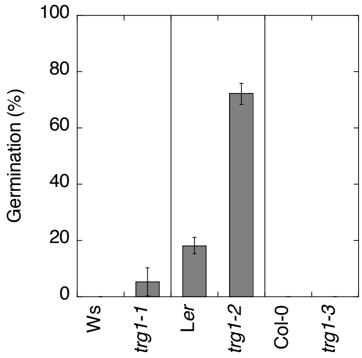

Supplementary Figure S4. Seed germination of *TRG1* alleles in red-light and far-red light conditions. The after-ripened seeds were imbibed for 1h before the light irradiation. The seeds were imbibed for five days in the dark as described in Methods. Means of three biological replicates with SE are shown for each genotype.

(A) Effect of imbibition temperature on seed germination of *TRG1* alleles under red-light pulse conditions.

(B) Effect of far-red light on germination of *trg1* mutant seeds imbibed at 22°C

A

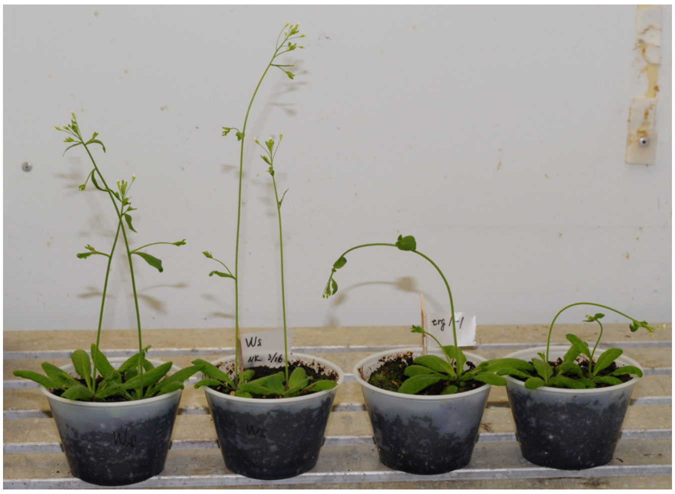

Ws (WT) *trg1-1*

B

Ws (WT) *trg1-1*

0h

1h

1.5h

2.5h

3.5h

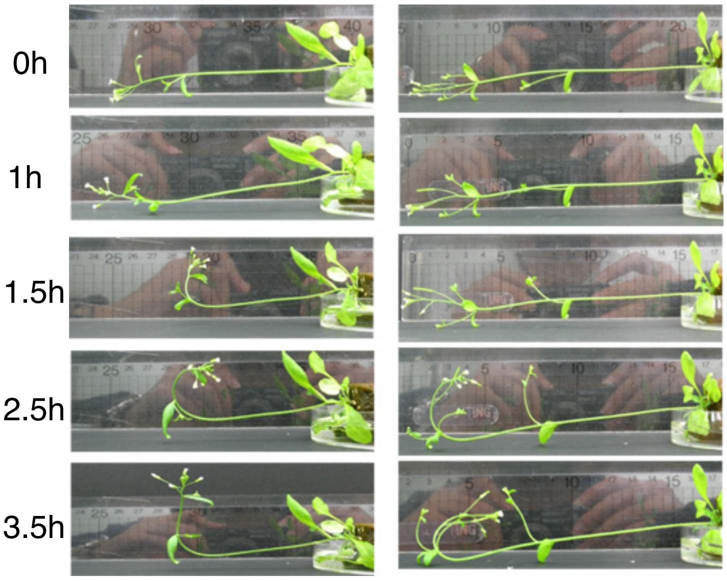

Supplementary Figure S5. Bending and gravitropic movement of *trg1-1* flower stem

(A) Plants at bolting stage.

(B) Time course of gravitropic response of the flower stem.

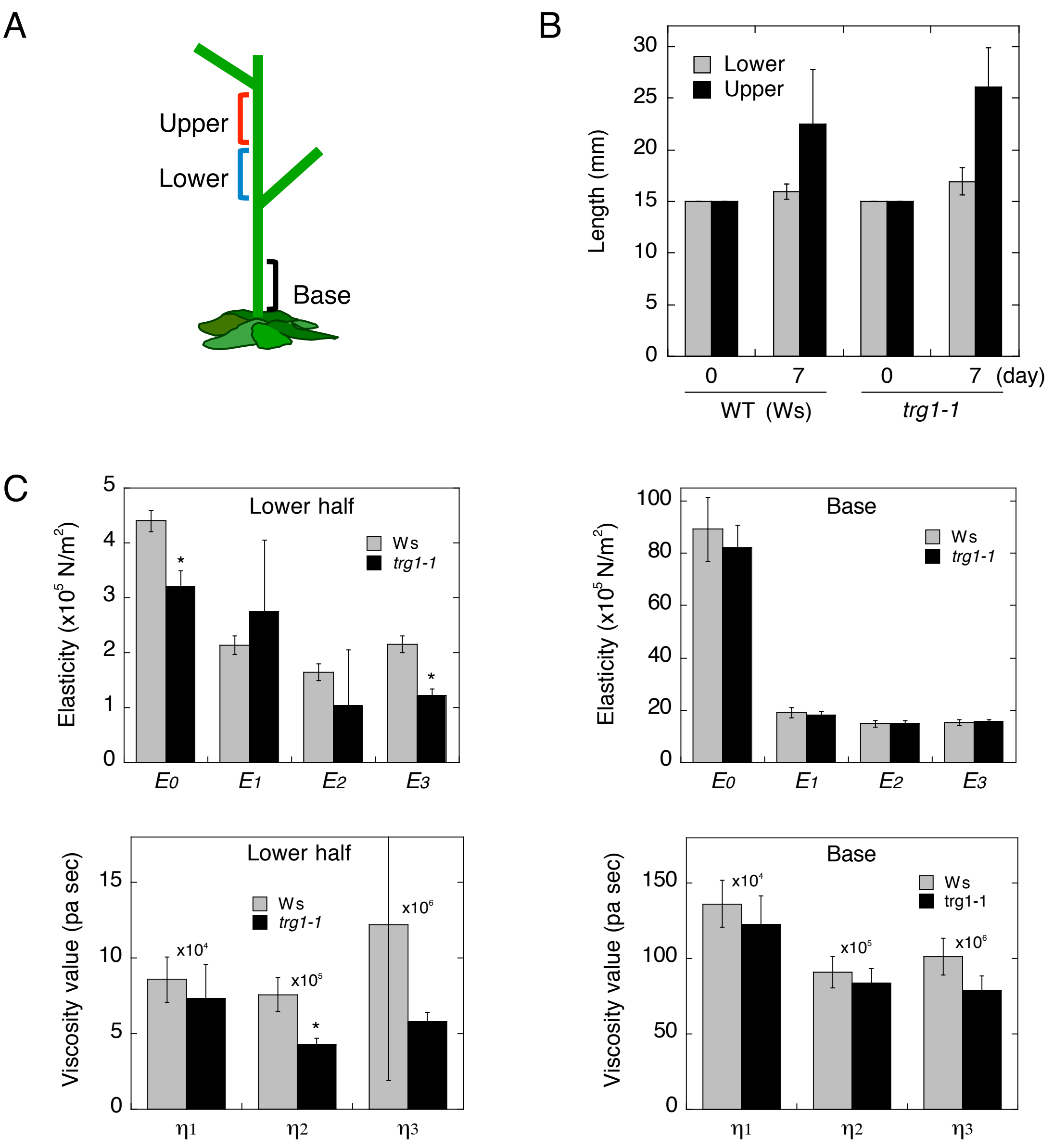

Supplementary Figure S6. Elongation of second internode halves and physical properties of non-elongating parts of the stem.
(A) Schematic representation of the internode segment positions used for elongation and creep-extension analyses.
(B) Elongation of upper and lower halves of the second internode during 7 days. When the second internode reached at 3cm in length (about 1 month after sowing), the internode was marked every 5mm, and the intervals between marks were measured after 7 days. Length of lower (gray) and upper (black) halves of wild type (Ws) and *trg1-1* are shown. Five plants were used for each genotype, and SE of the five biological replicates is shown as error bar.
(C) The lower-halves (1.5cm in length) of the second internode (3cm) and basal 1.5cm of the first internode were cut from the 1 month old plants and processed for creep-extension analysis. Elasticity and viscosity values of wild type (gray) and *trg1-1* (black) cell wall of the internode are shown. Five stem segments from five plants were used for each genotype, and SE of the five biological replicates is shown as error bar. Asterisks indicate statistical differences between wild type and mutant values ($P<0.05$, Student's *t* test).

A

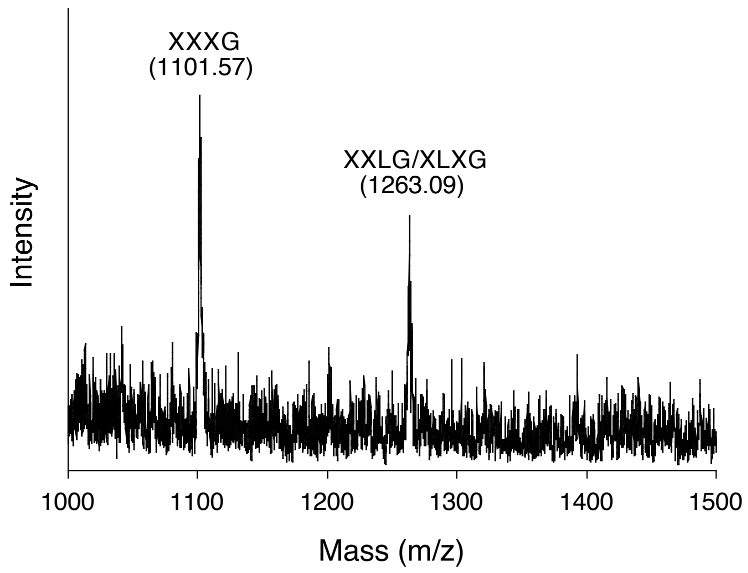

B

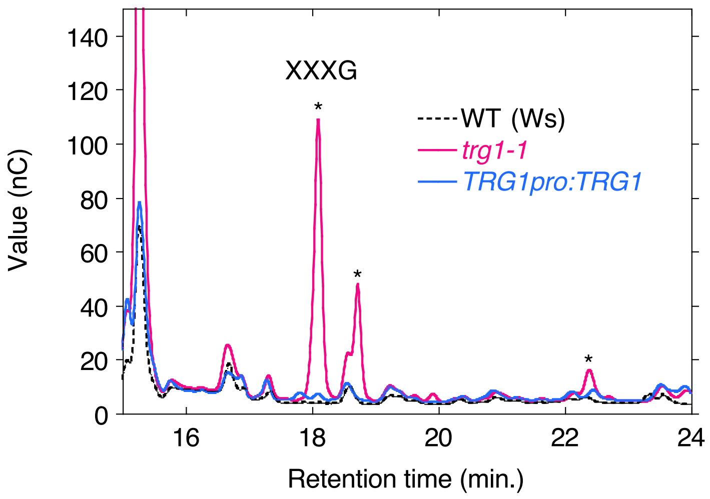

Supplementary Figure S7. HPLC and MALDI/TOF MS analyses of free oligosaccharides.

(A) Free oligosaccharides extracted from *trg1-1* fruits were analyzed by MALDI/ TOF MS. XXXG and XXLG/XLXG peaks were considered as potassium adducts.

(B) Free oligosaccharides were extracted from developing fruits. The extracts were analyzed by HPAEC with pulsed amperometric detection (PAD) . Asterisks denote *trg1-1* specific peak. XXXG standard was co-eluted with a major peak at 18.2 min.

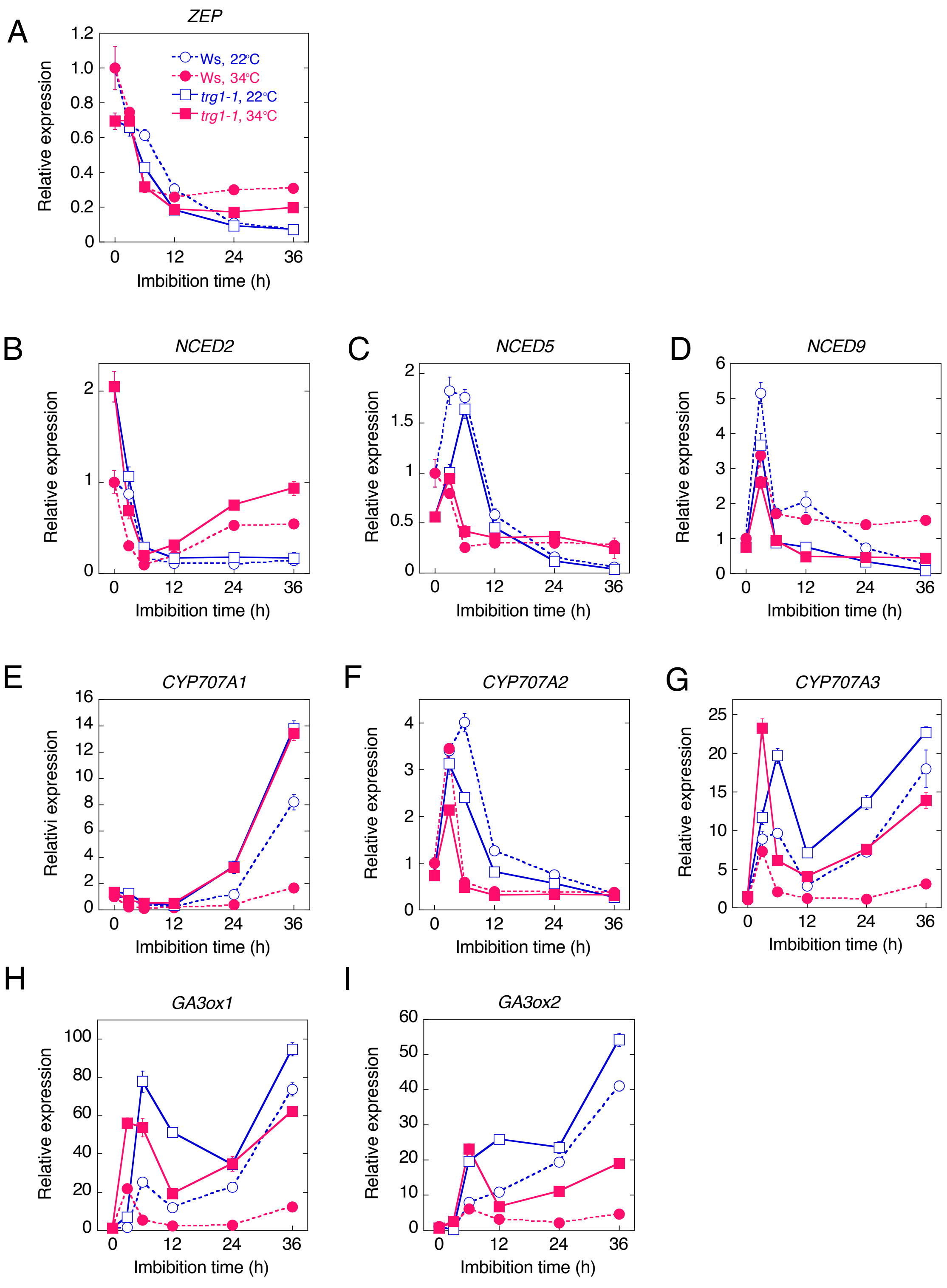

Supplementary Figure S8. Expression time course of ABA metabolism and GA biosynthesis genes in imbibed seeds.

Transcript levels were quantified by qRT-PCR. RNA was extracted from seeds imbibed for designated time to quantify each gene expression. Values are means of three technical replicates with SDs. RNA extraction and quantification analysis were repeated four times with different seed batches, and we obtained similar results from the different experiments. (A) *ZEP*, (B) *NCED2*, (C) *NCED5*, (D) *NCED9*, (E) *CYP707A1*, (F) *CYP707A2*, (G) *CYP707A3,* (H) *GA3ox1*, (I) *GA3ox2*. Relative values to WT dry seed level are plotted.
