# Supplementary material for: α-Xylosidase plays essential roles in xyloglucan remodelling, maintenance of cell wall integrity, and seed germination in Arabidopsis thaliana
Source: J Exp Bot. 2016 Sep 7;67(19):5615–29. doi: 10.1093/jxb/erw321 (PMC5066485; doi:10.1093/jxb/erw321)
Supplement: Supplementary Data [file supp_erw321_Supplementary_Tables_S1_S5.pdf]

Supplementary Table S1. Molecular markers used for *trg1* mapping

Marker name	Primer Sequence (mer)	Chr.	Marker type	Position (bp)	Anealing temp. (°C)	Restriction enzyme	Estimated size on a gel (bp)		Agarose conc. (%)
							Ws	Col+	
nga63	AACCAAGGCACAGAAGCG (18)	1	SSLP	3,244,463	55		92	118	5
	ACCCAAGTGATCGCCACC (18)			3,244,573					
nga248	TCTGTATCTCGGTGAATTCTCC (22)	1	SSLP	9,887,359	55		92	109	5
	TACCGAACCAAAACACAAAGG (21)			9,887,503					
nga280	CTGATCTCACGGACAATAGTGC (22)	1	SSLP	20,877,364	55		92	109	5
	GGCTCCATAAAAAGTGCACC (20)			20,877,467					
F5I14-49495	CTGCCTGAAATTGTCGAAAC (20)	1	SSLP	24,374,008	55		295	200	3
	GGCATCACAGTTCTGATTCC (20)			24,374,203					
14G4	CGTCACCGTTGCCACTTCCGCC (22)	1	CAPS	25,567,517	65	*ScrFI*	465	340, 105	2
	CACATATATGGCCACAACTCTCTGTAG (27)			25,567,942					
TJ-5	CTTCTCGCCATAGCTTCA (18)	1	INDEL	25,697,281	53		190	176	4
	CAGATCCGTCAATAACAG (18)			25,697,452					
FJ-4	TTCCGACCAACCGTTTCT (18)	1	CAPS	25,755,578	55	*BamH*I	405, 350, 210	565, 405	2
	AAGCCTTACACGCTAACA (18)			25,754,557					
FN-1	ATAATTCTTGTCTGTTGG (18)	1	INDEL	25,982,118	45		1,100	810	1
	AAGTGACGAAGATTAACA (18)			25,982,885					
KNAT2	GGTAGCCATATCAGTTCATTG (21)	1	CAPS	26,585,436	55	*AluI*	216, 175, 194, 62	410, 170, 62	4
	ATTTAGTTGACACATCAGCTATC (23)			26,586,066					
nga111	TGTTTTTTAGGACAAATGGCG (21)	1	SSLP	27,356,874	55		142	124	4
	CTCCAGTTGGAAGCTAAAGGG (21)			27,357,002					
m246	GCTTGAACTCCTCCTCCTTC (20)	2	CAPS	1,130,867	55	*MaeIII*	1122, 232	1450	0.8
	TGAAGAGCTATCCGAGATGG (20)			1,132,341					
nga168	TCGTCTACTGCACTGCCG (18)	2	SSLP	16,298,919	55		140	157	5
	GAGGACATGTATAGGAGCCTCG (22)			16,299,069					
nga162	CATGCAATTTGCATCTGAGG (20)	3	SSLP	4,608,284	55		88	107	5
	CTCTGTCACTCTTTTCCTCTGG (22)			4,608,390					
nga6	TGGATTTCTTCCTCTCTTCAC (21)	3	SSLP	23,042,025	55		160	146	5
	ATGGAGAAGCTTACACTGATC (21)			23,042,167					
nga8	GAGGGCAAATCTTTATTTCGG (21)	4	SSLP	5,628,810	55		165	150	5
	TGGCTTTCGTTTATAAACATCC (22)			5,628,966					
nga1107	GCGAAAAAACAAAAAAATCCA (21)	4	SSLP	18,096,131	55		139	148	5
	CGACGAATCGACAGAATTAGG (21)			18,096,282					
nga151	GTTTTGGGAAGTTTTGCTGG (20)	5	SSLP	4,669,932	55		102	146	5
	CAGTCTAAAAGCGAGAGTATGATG (24)			4,670,081					
nga76	GGAGAAAATGTCACTCTCCACC (22)	5	SSLP	10,418,614	55		198	234	5
	AGGCATGGGAGACATTTACG (20)			10,418,844					

Supplementary Table S2. Primers used for *TRG1/XYL1* (At1g68560) and *trg1-1* mutant allele cloning by PCR

Clone Name	Forward primer	Reverse primer	Appendix
TRG1/trg1	ACAATACACTTGCCTATTAGTGTT	TCATATCCTGAGACTGTCCTACCC	For identification of the mutation
TRG1pro:TRG1	CACCGATTCGTCGGCTACGTGGTAT	TGGATCTTCTGGGCTGGACTT	For complementation analysis, transform *trg1-1*
TRG1pro:GUS	CACCGATTCGTCGGCTACGTGGTAT	CATGGCTATAGAGTGGTACTTTA	For gene expression analysis, transform Ws

CACC sequence underlined is attached to the 5' end of gene specific sequence for cloning with pENTR Directional TOPO vector of Invitrogen.

Supplementary Table S3. Primers used for *TRG1/XYL1*and *trg1-1* sequencing

Primer name	Sequence
U-59	CATTGTCTGGTTTTCTGGCTTGGG
U493	TGCTCAATGCAAACTTACCTTC
U1061	CATACCAAGAGCGTTACGAGCTGA
U1603	CAGGACCTTCCCCACTTGGACAC
U2211	CAAGAACCGTGGTCCATGTCT
U2739	ACATACCTAAAGACCAGTACGGCA
U3304	GACTTGTGGTGGTTGTTCTCGTGGG
U3713	CCATGGCTATAGAGTGGTACTT

Supplementary Table S4. Primers for gene expression analysis with qRT-PCR

AGI code	Gene name	Sequence	Reference
Reference genes			
At2g20000	*HBT*	GTATAGCTCCACCACCACTT	Graeber et al., 2011
		TCTTCTAGGTGCTTGAAGAGT	
At2g28390	SAND family	AACTCTATGCAGCATTTGATCCACT	Czechowski et al., 2005
		TGATTGCATATCTTTATCGCCATC	
At4g34270	TIP41-like	GTGAAAACTGTTGGAGAGAAGCAA	Czechowski et al., 2005
		TCAACTGGATACCCTTTCGCA	
At5g15710	F-box protein	TTTCGGCTGAGAGGTTCGAGT	Czechowski et al., 2005
		GATTCCAAGACGTAAAGCAGATCAA	
At2g01010, At3g41768	*18S rRNA*	CAGATACCGTCCTAGTCTCAACCA	
		CAGCGGAGTCCTATAAGCAACAT	
ABA biosynthesis and catabolism			
At5g67030	*ABA1/ZEP*	TTGTTTGGCCGTAGTGAAGCT	
		AGACTCGATATCCGCTGGTATAAAA	
At4g18350	NCED2	GCGGCTGAGCGTGCATTAA	
		GGGAATAATTCCCGGCAATCT	
At1g30100	*NCED5*	CCTCCGTTAGTTTCACCAACACT	
		GGTGTGTCGGAGACGGAGTT	
At1g78390	*NCED9*	GGAAAACGCCATGATCTCACA	
		AGGATCCGCCGTTTTAGGAT	
At4g19230	*CYP707A1*	CTCACTCTCTTCGCCGGAAG	
		GGAGGGAGTGGGAGTTTGGAA	
At2g29090	*CYP707A2*	CGTCTCTCACATCGAGCTCCTT	
		GAGGGTGTTGATGGACTTTTGG	
At5g45340	*CYP707A3*	CTCTGTTTCTCTGTTTACTCCCGATTTA	
		CGTATCTTCTCTGTTTTGCTGCA	
GA biosynthesis			
AT1G15550	*GA3ox1*	GGACAAACCGGGTAGTGATTT	
		ATGGGCCAGTCTCAGTTCAG	
At1g80340	*GA3ox2*	GTTTCCAAGTGAGAGATGGGTAG	
		TCTCCACTTCCCAAACTGGT	
α-xylosidase			
AT1G68560	*TRG1/XYL1*	TTAGCCTCGTCATTGCTTTCC	
		TCCCGGTGGCGTAACCT	

Supplementary Table S5. Primers used for the confirmation of clone sequence for complementation and tissue specific gene expression analyses

Clone Name	Primer Name	Sequence
TRG1pro:TRG1	68560-L1351	CAGCTATCAATCTTAACACTGACC
	68560-L-184	GGTCATCGCATTAATTCTCAAACAAC
	68560-L1907	GAGTATCTGATTTTCCGACACAAC
	68560-L2574	GTCCGGTTTACTTCCCTGATTT
	68560-L3097	GAACTTCGGTATATTTGGAGTTCC
	68560-L3609	CCAAAACACTATCTTACCAACGCA
	68560-L556	CCATAAGCTAAAGAGGGTTCGGTT
	68560-L-613	CGAGATGTTACATGCATGTATG
	68560-L825	CTACGGCTCTGATATCACCACT
	68560-U1061	CATACCAAGAGCGTTACGAGCTGA
	68560-U1603	CAGGACCTTCCCCACTTGGACAC
	68560-U2211	CAAGAACCGTGGTCCATGTCT
	68560-U2739	ACATACCTAAAGACCAGTACGGCA
	68560-U4265	GTATTGTAGTGATCTATCATCTATC
	68560-U4794	AATATATACTCCGTGTCCGTGAC
	68560-U493	TGCTCAATGCAAACTTACCTTC
	68560-U5326ws	AACAGTATAAATTTTTAACGGTACAC
	68560-U-551	ACCGGTTTGTCTTCCTAGAGT
	68560-U-775	ATGCATTCCTCACCTGGCTTC
TRG1pro:GUS	GUS-L1222	GATGTGGAGTATTGCCAACG
	GUS-L1540	GCTGCACTCAATGTACACC
	GUS-L415	ACTGAACTGGCAGACTATCC
	GUS-L880	CTTTGGTCGTCATGAAGATGC
	GUS-U309	CTGATGCTCCATCACTTCCT
TOPO Vector	M13 forward primer	GTAAAACGACGGCCAG
	M13 reverse primer	CAGGAAACAGCTATGAC
pGWB Vector	attB1	ACAAGTTTGTACAAAAAAGCAGGCT
	attB2	CACTTTGTACAAGAAAGCTGGGT
